# Effects of Different Correlation Metrics and Preprocessing Factors on Small-World Brain Functional Networks: A Resting-State Functional MRI Study

**DOI:** 10.1371/journal.pone.0032766

**Published:** 2012-03-06

**Authors:** Xia Liang, Jinhui Wang, Chaogan Yan, Ni Shu, Ke Xu, Gaolang Gong, Yong He

**Affiliations:** 1 State Key Laboratory of Cognitive Neuroscience and Learning, Beijing Normal University, Beijing, China; 2 Department of Radiology, The First Affiliated Hospital, China Medical University, Shenyang, China; Cuban Neuroscience Center, Cuba

## Abstract

Graph theoretical analysis of brain networks based on resting-state functional MRI (R-fMRI) has attracted a great deal of attention in recent years. These analyses often involve the selection of correlation metrics and specific preprocessing steps. However, the influence of these factors on the topological properties of functional brain networks has not been systematically examined. Here, we investigated the influences of correlation metric choice (Pearson's correlation versus partial correlation), global signal presence (regressed or not) and frequency band selection [slow-5 (0.01–0.027 Hz) versus slow-4 (0.027–0.073 Hz)] on the topological properties of both binary and weighted brain networks derived from them, and we employed test-retest (TRT) analyses for further guidance on how to choose the “best” network modeling strategy from the reliability perspective. Our results show significant differences in global network metrics associated with both correlation metrics and global signals. Analysis of nodal degree revealed differing hub distributions for brain networks derived from Pearson's correlation versus partial correlation. TRT analysis revealed that the reliability of both global and local topological properties are modulated by correlation metrics and the global signal, with the highest reliability observed for Pearson's-correlation-based brain networks without global signal removal (WOGR-PEAR). The nodal reliability exhibited a spatially heterogeneous distribution wherein regions in association and limbic/paralimbic cortices showed moderate TRT reliability in Pearson's-correlation-based brain networks. Moreover, we found that there were significant frequency-related differences in topological properties of WOGR-PEAR networks, and brain networks derived in the 0.027–0.073 Hz band exhibited greater reliability than those in the 0.01–0.027 Hz band. Taken together, our results provide direct evidence regarding the influences of correlation metrics and specific preprocessing choices on both the global and nodal topological properties of functional brain networks. This study also has important implications for how to choose reliable analytical schemes in brain network studies.

## Introduction

Resting-state functional MRI (R-fMRI) has recently emerged as a powerful tool for exploring spontaneous brain function [1]. The low-frequency (<0.1 Hz) fluctuations in the fMRI signal temporally interact across functionally related areas, collectively constituting a complex neural network, which has been referred to as “functional connectome” [2], [3]. Analyses of functional brain networks based on graph theory have revealed many non-trivial topological properties, such as small-world attributes (high local clustering and short path lengths [4]) [5], high efficiency at a low wiring cost [6] and highly connected hubs [7], [8], [9], [10]. Graph-based analysis has also been used to investigate the topological changes of functional brain networks under pathological conditions [3], [11], [12], [13]. These studies have shaped our understanding of how the functional brain network is topologically organized under both healthy and diseased states.

In general, a brain network is composed of two basic elements: nodes and edges. In R-fMRI-based brain networks, nodes usually represent brain entities (e.g., brain regions), and edges represent functional associations between the brain entities. How to define nodes and edges are two core questions in graph-based network analysis [3], [12], [14]. Recent studies have demonstrated that different nodal definitions lead to different results in brain network analysis [15], [16], [17], [18]. Likewise, there are similar concerns regarding the effects of edge definitions on the topological properties of brain networks. Different functional connectivity metrics have been used to define network edges in fMRI data analysis, including Pearson's correlation [8], [10], [17], [19] and partial correlation [20], [21], [22]. The former measures the general dependence between variables, whereas the latter estimates the direct interdependence after ruling out third-party effects [23], [24]. Recently, Smith et al. [25] demonstrated that both correlation methods provide excellent performance at estimating functional connections, but Pearson's correlation outperformed partial correlation when the number of nodes in brain networks significantly increased. Of note, most fMRI-based brain network studies have focused on only one of the two correlation metrics, and it remains unclear how the use of these different correlation metrics influences the topological properties of brain functional networks.

Another major concern in R-fMRI studies is related to preprocessing procedures. For example, some studies have removed global brain signals by regression to reduce confounding physiological effects [26], [27], [28], [29]. However, the validity of global signal regression in fMRI studies is still under debate [30], [31]. Recently, Weissenbacher et al [32] has quantitatively evaluated the impact of global signal regression on resting-state functional connectivity and found approximately doubled specificity of connections and widespread artificial anticorrelations after global signal regression. In the literature of functional brain networks, using R-fMRI, several research groups have constructed brain functional networks by regressing out the global signal [8], [10], [17], whereas others have not [7], [15], [20], [33]. A recent R-fMRI study has demonstrated that global signal removal alters topological structure of brain functional networks [34]. However, it remains unclear how the removal of global signals affects the topological properties of Pearson's-correlation-based and partial-correlation-based brain networks.

Another preprocessing factor that likely affects R-fMRI analysis is the use of different temporal filtering frequency bands. Buzsáki and colleagues [35], [36] observed that the center and range of different brain oscillation bands are distributed linearly on the natural logarithmic scale, suggesting that each band serves a different physiological function. Using R-fMRI, Salvador et al. [37], [38] found that the functional connectivity among brain regions depends on different frequency bands within the detectable frequency range. Moreover, functional brain networks derived from R-fMRI data exhibit differential small-world attributes across different frequency bands [6], [7], [39]. However, direct, detailed comparisons are needed to elucidate the influences of different frequency bands on the topological analysis of functional brain networks.

In consideration of these factors, relevant questions include how to choose among these different processing strategies and which combination of these factors provides the most appropriate descriptions for modeling R-fMRI-based brain networks. Given that no ‘golden standard’ currently exists, in this study, we sought to answer this question by assessing the test-retest (TRT) reliability of network analyses while varying processing factors, assuming that a ‘better’ network analytic strategy will produce a more reliable network structure. Although several recent studies have examined the TRT reliability of structural [40], [41] and functional [42], [43], [44], [45] brain networks, the effects of these processing factors on R-fMRI brain networks remain to be further elucidated.

In this study, we aimed to systematically investigate i) whether topological structures of brain functional networks derived from R-fMRI data are significantly influenced by varying processing choices (e.g., different correlation metrics: Pearson's correlation versus partial correlation; with and without global brain signal regression; different frequency bands) and ii) which R-fMRI processing strategy produces the most reliable topological structure across short-term (<1 h apart) and long-term (>5 months apart) scans. To address these questions, we first used a public R-fMRI dataset with 22 participants (http://www.nitrc.org/projects/fcon_1000/) (dataset 1) to construct functional brain networks under different processing strategies, and we then compared the topological properties of the resultant networks using paired statistical tests. Finally we employed another public R-fMRI dataset with 25 participants (http://www.nitrc.org/projects/trt) (dataset2) to evaluate the short- and long-term TRT reliability of brain networks derived from these different processing choices.

## Materials and Methods

### Data Acquisition

#### Dataset1

Dataset1 was selected from a large sample R-fMRI dataset that was publicly released as a part of the 1000 Functional Connectomes Project (http://www.nitrc.org/projects/fcon_1000/) [2]. This dataset includes 22 right-handed healthy volunteers (20.1±1.67 years, 11 males) with no history of neurological or psychiatric disorders. Written informed consent was obtained from each participant, and the protocols were approved by the Ethics Committee of the Institutional Review Board of the Imaging Center for Brain Research, Beijing Normal University. All of the subjects were scanned in a 3.0 Tesla SIEMENS MR scanner at Beijing Normal University Imaging Center for Brain Research. The functional images were obtained using an echo-planar imaging sequence with the following parameters: 33 axial slices, thickness/gap = 3/0.6 mm, in-plane resolution = 64×64, time repetition = 2000 ms, time echo = 30 ms, flip angle = 90° and field of view = 200×200 mm2. During the resting-state session, the participants were instructed to hold still, keep their eyes closed, stay awake and not think of anything in particular. According to a simple questionnaire administered after the scan, none of the participants fell asleep during the scan.

#### Dataset2

Dataset2 is a TRT R-fMRI dataset with 25 participants (30.7±8.8 years, 9 males) that is publicly available at NITRC (http://www.nitrc.org/projects/trt). All participants had no history of psychiatric or neurological illness. Informed consent was obtained prior to participation. Data collection was carried out according to protocols approved by the institutional review boards of New York University (NYU) and the NYU School of Medicine. Three resting-state scans were obtained for each participant using echo-planar imaging sequence on a Siemens Allegra 3.0-Tesla scanner with the following parameters: 39 axial slices, in-plane resolution = 64×64, time repetition = 2000 ms, time echo = 25 ms, flip angle = 90°, field of view = 192×192 mm2. Scans 2 and 3 were conducted in a single scan session, 45 min apart, 5–16 months (mean 11±4) after scan 1. All individuals were asked to relax and remain still with their eyes open during the scan.

### Data Preprocessing

**Figure 1:**
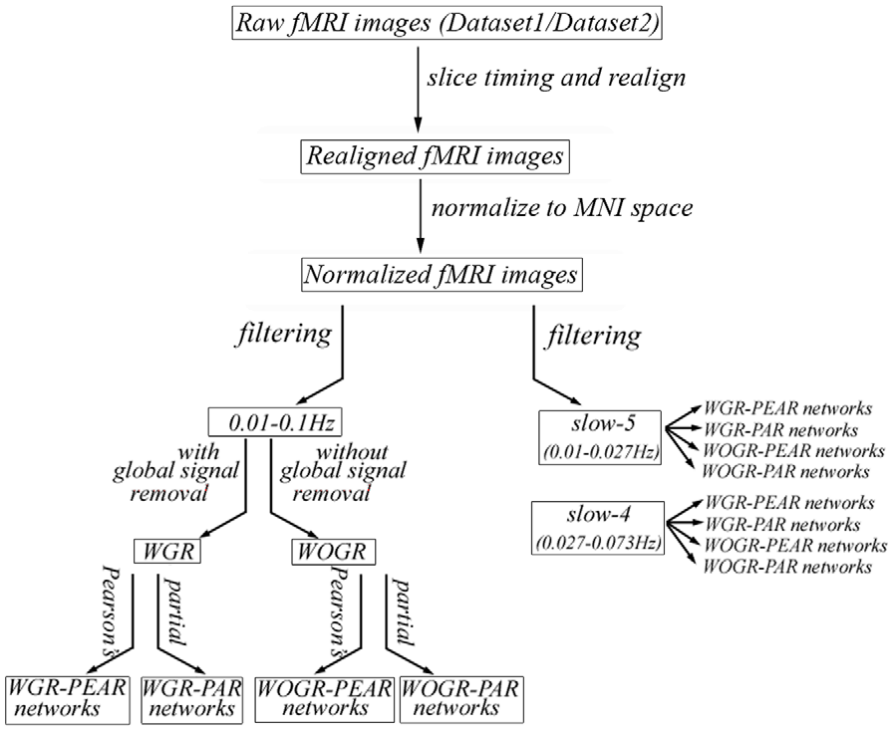
A flowchart for varying processing strategies prior to brain network construction.

Both dataset1 and dataset2 were preprocessed as follows: the first 10 volumes were discarded to allow the MRI signal to reach a steady state and allow the subjects to adapt to the scanner noise. We used the statistical parametric mapping package (SPM5, http://www.fil.ion.ucl.ac.uk/spm) to perform image preprocessing as follows. First, functional images were corrected for the acquisition time delay between slices of each volume and for head motion between volumes using a six-parameter (rigid body) spatial transformation. The resulting images were further spatially normalized to the Montreal Neurological Institute (MNI) EPI template and resampled into 3-mm isotropic voxels. Given the widely used frequency interval of 0.01–0.1 Hz in the R-fMRI literature [1], [46], the preprocessed data were band-pass filtered within this primary band to reduce the effects of low frequency drift and high-frequency physiological noises. To investigate the impact of different frequency bands, we also applied Buzsaki's nomenclature [35] to divide the whole frequency spectrum into four different frequency intervals: slow-5 (0.01–0.027 Hz; centered at 0.0185 Hz), slow-4 (0.027–0.073 Hz; centered at 0.05), slow-3 (0.073–0.198 Hz; centered at 0.14 Hz) and slow-2 (0.198–0.25 Hz; centered at 0.22 Hz). This division has been used in several previous R-fMRI studies [47], [48], [49]. We restricted our analysis to the slow-5 and slow-4 bands because the other bands mainly reflect high-frequency physiological noises. Network analysis was performed for the two bands in the same manner as for the primary broadband (0.01–0.1 Hz) analysis (for a flowchart of the data process, see Fig. 1).

### Correlation Matrix and Graph Construction

We defined network nodes by parcellating the brain into 90 regions of interest (ROIs) according to the Anatomical Automatic Labeling atlas (AAL) [50] (Table S1). A representative mean time series for each region was extracted by averaging the time series of all voxels within that region followed by multiple linear regression analysis to remove head motion profiles.

#### Global signal regression

To evaluate the effects of global signal regression on network structure, we obtained two sets of time courses, one acquired using linear regression of global signals and the other acquired without it.

#### Correlation metrics

The two sets of time series were then used to measure functional connectivity among regions by calculating Pearson's correlation coefficients and partial correlation coefficients between any possible pair of regional time series. The mean time course for each subject can be denoted as 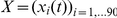, where 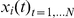 is the mean time series of the 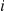 ^th^ region. The two connectivity metrics are calculated as follows:

Pearson's correlation:
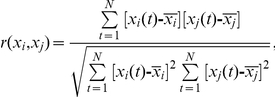
where 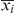 denotes the average of 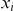.

Partial correlation:
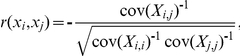
where 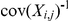 represents the 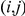
th element in the inverted covariance matrix from the data in matrix 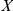. Note that in partial correlation analysis, the length of the time series should be larger than the number of regions because the covariance matrix needs to be inverted; if not, the inversion can be numerically unstable. In the present analysis, we have more time series (N = 230 for dataset1 and N = 184 for dataset2) than regions (n = 90), and the inversions of partial correlation were stable all through the analysis.

Combination of the two sets of time series (global-signal regressed or not) with the two correlation methods (Pearson's correlation and partial correlation) resulted in four correlation matrices for each participant: 1) Pearson's-correlation-based functional connectivity estimation on data with global-signal regression (WGR-PEAR); 2) Partial-correlation-based functional connectivity estimation on data with global-signal regression (WGR-PAR); 3) Pearson's-correlation-based functional connectivity estimation on data without global-signal regression (WOGR-PEAR); and 4) Partial-correlation-based functional connectivity estimation on data without global-signal regression (WOGR-PAR).

All of the four types of correlation matrices derived above were further thresholded into binary networks. We chose network sparsity (S) (the number of existing edges divided by the maximum possible number of edges) as the threshold measurement. The sparsity threshold makes two groups of networks comparable by normalizing the number of edges among all of the networks and excluding the effects of low-level correlation discrepancies on topological architecture. Given the lack of a definitive way to select a single threshold, a continuous range of 0<S<1 was employed to threshold the correlation matrices into a set of binary matrices (i.e., networks).

Given that binary graphs neglect detailed information that may bias the topological analysis, we also generated weighted brain networks over the whole range of sparsity (0<S<1) from correlation matrices to explore the influences of the factors studied here (for details, see Text S1

### Network Analysis

We investigated the topological properties of brain networks at both the global and regional level. At the global level, we focused on small-world parameters [clustering coefficient (Cp), characteristic path length (Lp), normalized clustering coefficient (γ), normalized characteristic path length (λ) and small-worldness (σ)], network efficiency [global efficiency (E_glob_) and local efficiency (E_loc_)], assortativity (α) and hierarchical topology (β). At the regional level, we computed the degree of centrality (k) for each brain region and employed the measure to identify network hubs because k is the most reliable nodal measurement [44]. To provide a threshold-independent network assessment, we calculated the area under the curve (AUC, i.e., the integral) for each network metric (both global and nodal). The integrated network metrics were used to perform further statistical comparisons and TRT reliability analysis. Network hubs were also identified by using the integrated degree values. The network parameters used in the present study are summarized in Table 1. For details about the computation of network parameters, see Text S1 and [51].

**Table 1:** Topological parameters of brain functional networks used in this study.

Parameters	Character	Descriptions	Range
***Global network properties***			
**Clustering coefficient**	*C_p_*	The capability of local clustering of a network	[0, 1], where 1 means full clustering
**Characteristic path length**	*L_p_*	The extent of overall routing efficiency of a network	[1,∞), where 1 for fully connected network, ∞ for network with disconnected nodes
**Gamma**	γ	The normalization of *C_p_* divided by those of comparable random networks	(0, ∞)
**Lambda**	λ	The normalization of *L_p_* divided by those of comparable random networks	(0, ∞)
**Sigma**	σ	The small-worldness indicating the extent of a network between randomness and order	(0, ∞)
**Local efficiency**	*E_loc_*	How efficient of information transfer over a node's direct neighbors	[0, 1]
**Global efficiency**	*E_glob_*	How efficient of information transfer through the whole network	[0, 1]
**Assortativity**	_α_	The tendency of nodes to link with those nodes with similar number of edges	[−1, 1], where positive means assortative
**Hierarchy**	_β_	The tendency of self-similar nesting of different groups or modules into each other	(−∞,∞), where positive means hierarchy
***Regional nodal properties***			
**Degree**	*k_i_*	The number of connections linked directly to a node	[0, N-1], N is the number of nodes

### Statistical Analysis

To determine the impact of each of the three processing factors (correlation metric, global signal regression and frequency band) on integrated global network parameters (Cp, Lp, 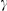, 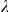, 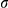, E_loc_, E_glob_, 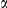 and 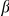) and regional nodal property (k), nonparametric paired-sample Wilcoxon signed rank tests were performed on dataset1 in the following manner: 1) To evaluate the influence of the correlation measure, statistical comparisons were made between Pearson's-correlation-based networks and partial-correlation-based networks (i.e., WGR-PEAR vs. WGR-PAR and WOGR-PEAR vs. WOGR-PAR). 2) To evaluate the influence of removing global signals, statistical comparisons were made between networks with and without global signal regression in the case of Pearson's correlation metric (i.e., WGR-PEAR vs. WOGR-PEAR) because removing global signals is inherent to the partial correlation metric and makes the global signal an insignificant factor. The Bonferroni correction was used for multiple comparisons and the significance level set at p<0.017 (0.05/3). Of note, given the commonly used frequency band of 0.01 to 0.1 Hz in the existing literature of R-fMRI studies and for simplicity, the effects of the correlation metrics and global signals were estimated using only datasets that were band-filtered in the specific frequency interval of 0.01 to 0.1 Hz. 3) To evaluate the influence of different frequency bands, statistical comparisons were made between networks at two different frequency bands (i.e., slow-5 vs. slow-4). These networks were separately constructed under the following four conditions: WGR-PEAR, WOGR-PEAR, WGR-PAR and WOGR-PAR (Fig. S1, S2, S3, S4).

### Test-Retest Reliability

To evaluate the TRT reliability of brain networks under different processing choices, we computed an intraclass correlation coefficient (ICC) [52] based on Dataset 2. For each global or nodal network measure derived under each combination of the three factors mentioned above, we obtained its short-term ICC between scans 2 and 3 and its long-term ICC between scan 1 and the average of scans 2 and 3. Of note, the averaging was done on individual functional connectivity matrices rather than graph metrics between scan 2 and scan 3 followed by computing graph metrics. The ICC has been defined as [52]:
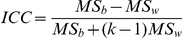
Where 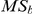 is the between-subject variance, 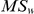 is the within-subject variance and k is the number of repeated observations per subject. ICC is close to 1 for reliable measures that show low within-subject variance relative to between-subject variance and 0 (negative) otherwise. In the current study, reliability was recorded in terms of the criteria of [53], [54], with an ICC value from 0 to 0.25 indicating poor, 0.25 to 0.4 indicating low, 0.4 to 0.6 indicating fair, 0.6 to 0.75 indicating good and 0.75 to 1.0 indicating excellent reliability. Because the network metrics were integrated over the entire threshold range, ICC is a single scalar for each network measure.

## Results

We generated both binary and weighted brain networks to evaluate the influences of processing factors on network topology and found similar results, indicating robust findings regardless of binary or weighted approach. Therefore, we only reported the results derived from binary networks here. For weighted results, see Supplemental figures (Fig. S5, Fig. S6, Fig. S7, Fig. S8, Fig. S9, Fig. S10, Fig. S11, Fig. S12).

### Robust small-world functional brain networks

**Figure 2:**
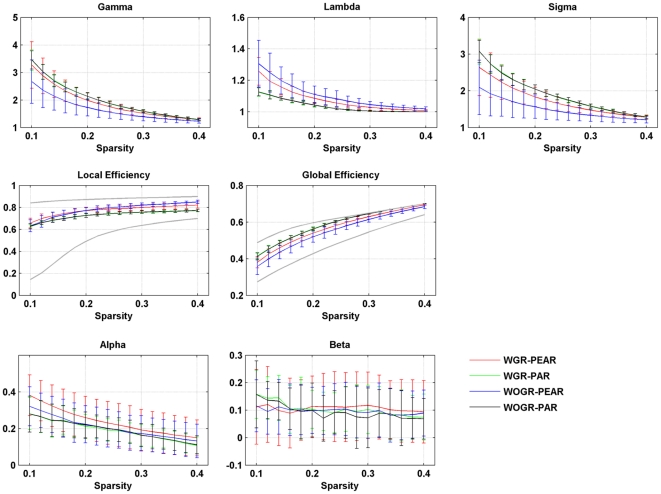
Global topological properties of Pearson's-correlation and partial-correlation-based networks with and without global signal regression. Plots show the changes in small-world parameters (Cp, Lp, γ, λ and σ), network efficiency (Local efficiency and Global efficiency), assortativity coefficient (α) and hierarchy coefficient (β) in functional brain networks dependent on both correlation metrics (Pearson's correlation or partial correlation) and global signal (regressed or not) as a function of sparsity thresholds. Local and global efficiency of random and regular networks with the same number of nodes and edges as the real networks were shown in gray lines in the network efficiency plots.

Graph theoretical analysis revealed that functional brain networks derived from R-fMRI data show prominent small-world architecture across a wide sparsity range, Figure 2 showed the global network parameters within a sparsity range from 0.1 to 0.4, where the networks are sparse and their small-world attributes are estimable [4]. Compared with random networks, brain networks are highly clustered (i.e., γ>1) and have approximately equivalent path lengths (λ∼1). We also compared the global and local efficiency of the brain networks with those of comparable random networks and regular lattices. The results show that the efficiency curves of the brain networks are generally intermediate between the two extreme cases: the brain networks have greater global efficiency than the lattices but less than the random networks, and they have greater local efficiency than the random networks but less than the lattices (Fig. 2). Taken together, our observations indicate that human functional brain networks have efficient small-world properties regardless of the correlation metric selected or the application of global signal regression. Furthermore, functional brain networks were found to be assortative (assortative coefficients 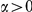) and hierarchical (hierarchy coefficients 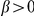) over a wide sparsity range (Fig. 2). Under the two subdivided frequency bands (i.e., slow-5 and slow-4), all of the above-mentioned global topological characteristics (small-world, network efficiency, assortative and hierarchy) were also found to be present in the functional brain networks constructed under all four different conditions: WGR-PEAR (Fig. S1), WGR-PAR (Fig. S2), WOGR-PEAR (Fig. S3) and WOGR-PAR (Fig. S4).

### Correlation metrics and global signal regression: dependence and reliability

#### Effects of correlation metrics

**Figure 3:**
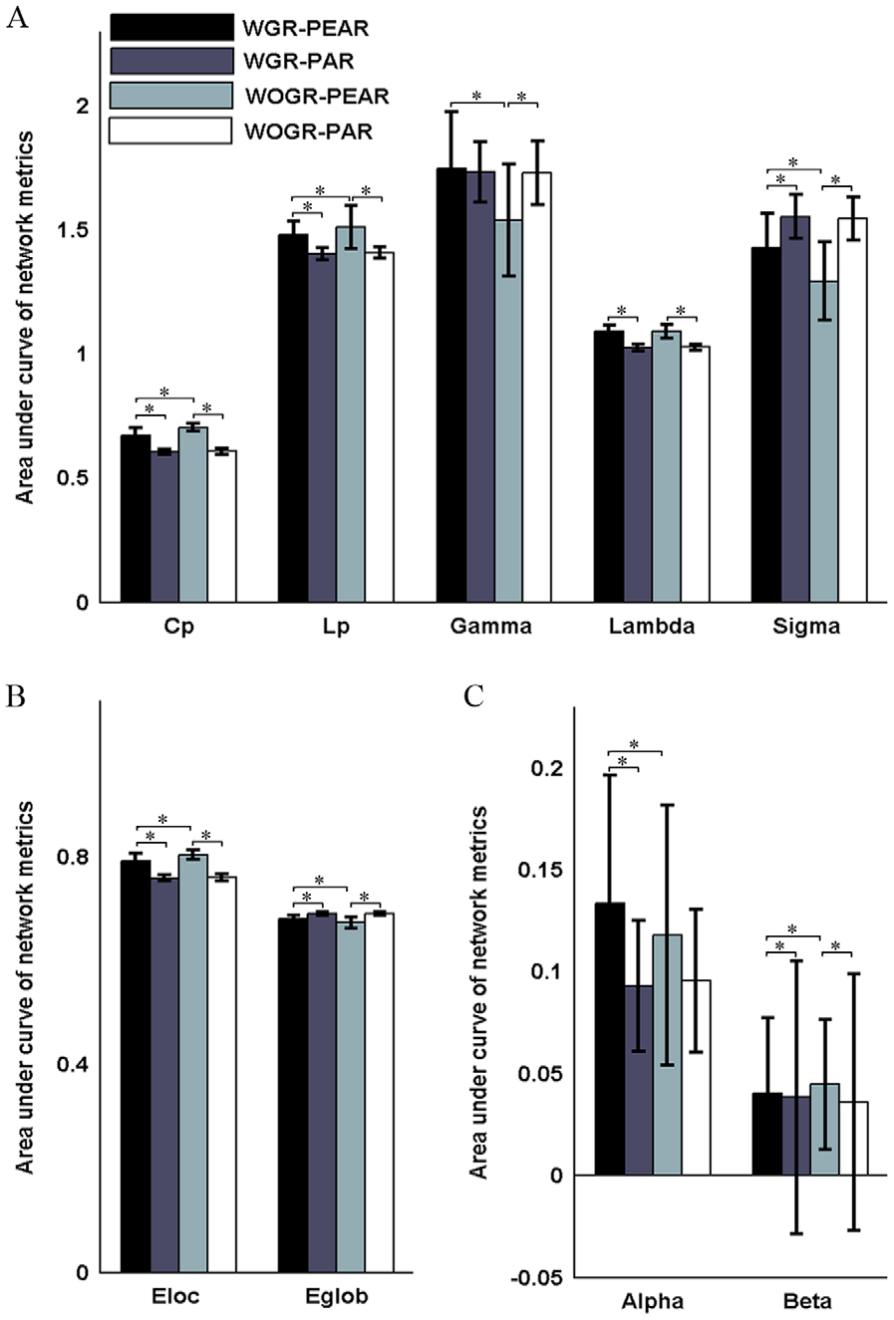
Correlation metrics and global signal dependent differences in global network properties. Bars show the differences in the areas under curves (AUC) of (A) small-world parameters (Cp, Lp, γ, λ and σ), (B) network efficiency (Local efficiency and Global efficiency) and (C) assortativity coefficient (α) and hierarchy coefficient (β). Error bars correspond to standard deviation of the mean across participants. The asterisk indicates p<0.05.

We examined the effects of the different correlation metrics on both the global and regional properties of functional brain networks derived from the widely-used frequency band ranging from 0.01 to 0.1 Hz (i.e., WGR-PEAR vs. WGR-PAR and WOGR-PEAR vs. WOGR-PAR). We found that, compared with partial-correlation-based networks, Pearson's-correlation-based networks had greater Cp (WGR-PEAR>WGR-PAR: p = 4.61e^−5^; WOGR-PEAR>WOGR-PAR: p = 4.01e^−5^) and E_loc_ (WGR-PEAR>WGR-PEAR: p = 4.61e^−5^; WOGR-PEAR>WOGR-PAR: p = 4.01e^−5^) but longer Lp (WGR-PEAR>WGR-PAR: p = 9.15e^−5^; WOGR-PEAR>WGR-PAR: p = 4.01e^−5^) and λ (WGR-PEAR>WGR-PAR: p = 4.01e^−5^; WOGR-PEAR>WOGR-PAR: p = 4.01e^−5^) and lower E_glob_ (WGR-PEAR<WGR-PAR: p = 4.61e^−5^; WOGR-PEAR<WOGR-PAR: p = 4.01e^−5^) (Fig. 3). When scaled to degree-matched random networks, differences in Cp between the WGR-PEAR and WGR-PAR networks disappeared in γ, which could be due to the effect of normalization to random networks with topological property magnitudes similar to those of the corresponding brain networks. Moreover, compared with partial-correlation-based networks, Pearson's-correlation-based networks with global signal regression are marginally more assortative (WGR-PEAR>WGR-PAR: p = 0.018), and they are more hierarchical regardless of global signal regression (WGR-PEAR>WGR-PAR: p = 0.0036; WOGR-PEAR>WOGR-PAR: p = 0.001) (Fig. 3).

**Figure 4:**
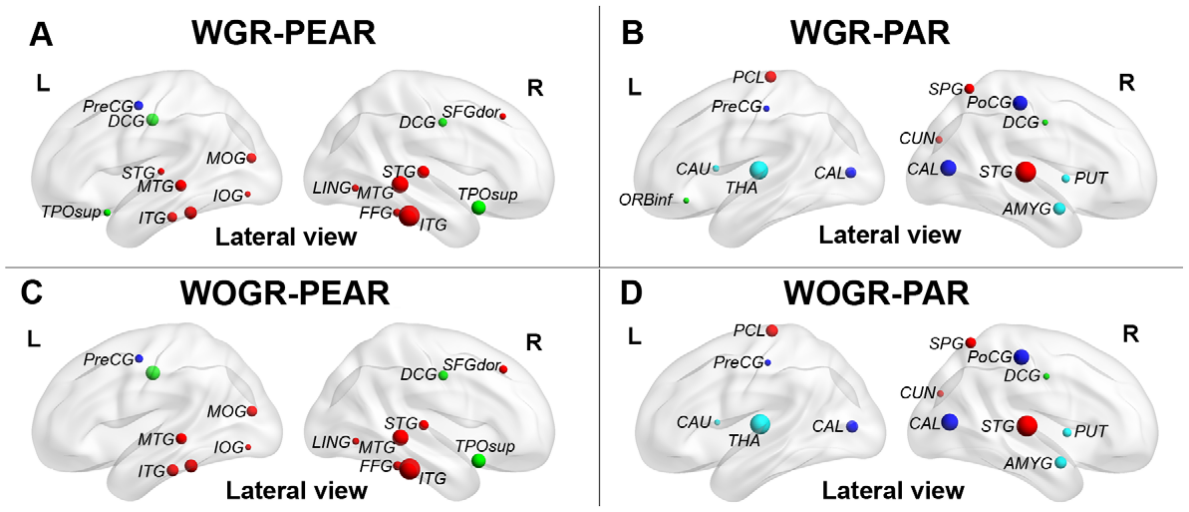
Functional hubs derived from networks using different correlation metrics and global signal strategies. (A) WGR-PEAR, (B) WOGR-PEAR, (C) WGR-PAR and (D) WOGR-PAR. Regions with degree>the mean+standard deviation were considered hubs. Node colors were coded according to their membership of classical cortex classifications: association cortex (red), limbic cortex (purple), paralimbic cortex (green), subcortical regions (light blue) and primary cortex regions (dark blue).

To explore influences on regional network architecture, we computed the degree of each node and then identified the network hub regions with the highest degree (for details, see Materials and Methods). Fig. 4 shows the nodal degree of all hub regions in WGR-PEAR, WGR-PAR, WOGR-PEAR and WOGR-PAR networks. As illustrated in Figure 4, brain networks derived from Pearson's versus partial correlation metrics appeared to have different hub distributions. In Pearson's-correlation-based networks (i.e., WGR-PEAR and WOGR-PEAR), the hubs are predominately located at several association cortical regions (e.g., the inferior temporal gyrus [ITG], the middle temporal gyrus [MTG] and the superior temporal gyrus [STG]) and paralimbic cortical regions (e.g., the superior temporal pole [TPOsup] and the middle cingulate gyrus [MCG]). However, in partial-correlation-based networks (i.e., WGR-PAR and WOGR-PAR), the network hubs are primarily located at regions in the primary cortex (e.g., the postcentral gyrus [PoCG] and calcarine [CAL]) and subcortical cortex (e.g., the thalamus [THA] and putamen [PUT]) with high degree (for details, see Fig. 4). Indeed, the correlation in nodal degree was very low between the Pearson's-correlation- and partial-correlation-based networks [r = 0.163 and p = 0.124 (WGR-PEAR vs. WGR-PAR); r = 0.176 and p = 0.09 (WOGR-PEAR vs. WOGR-PAR)].

#### Global signal effects

We next evaluated the influence of global signal regression on global topological parameters in Pearson's-correlation-based brain networks (i.e., WGR-PEAR vs. WOGR-PEAR). WOGR-PEAR networks have greater Cp (p = 5.3e^−5^), γ (p = 0.0001) and E_loc_ (p = 0.0002) values than WGR-PEAR networks (Fig. 3). Additionally, WGR-PEAR networks are more assortative (p = 0.018) and hierarchical (p = 0.005) than WOGR-PAR networks. Brain networks with global signals removed versus conserved had a relatively consistent spatial pattern of hubs primarily distributed in the association cortex (e.g., ITG, MTG and STG) and paralimbic cortex (e.g., TPOsup and MCG) (Fig. 4). The nodal degree of all regions in WGR-PEAR networks were highly correlated with those in WOGR-PEAR networks (r = 0.966 and p<2.5e-4).

#### TRT reliability of network metrics

**Figure 5:**
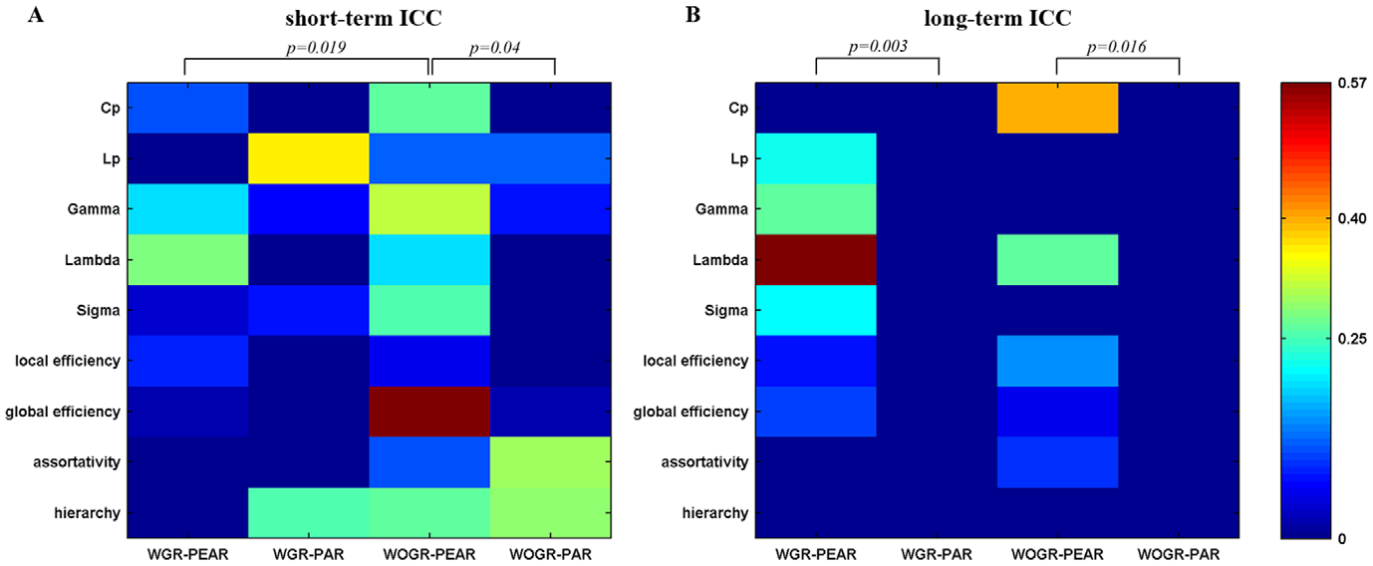
TRT reliability of global topological properties for Pearson's-correlation and partial-correlation-based networks with and without global signal regression. The reliability was estimated using areas under curves (AUC) of each metric. Statistical analysis revealed significant differences in (A) short-term and/or (B) long-term TRT reliability driven by correlation metrics and/or global signal regression.

Given that particular choices of processing options (i.e., correlation metrics and global signal regression) can make significant differences in network topological parameters, we next asked which analytical scheme would perform the best at modeling brain networks from the perspective of TRT reliability. Figure 5 shows the TRT reliability of 9 global network metrics under four different processing choices. Generally, most global network metrics exhibited poor to low reliability irrespective of the correlation metric or global signal regression. To test whether there is a difference in the TRT reliability associated with different processing options, we further performed a nonparametric paired-sample Wilcoxon signed rank test on the ICCs of global graph metrics. Our results showed that global graph metrics derived from Pearson's-correlation-based networks are more reliable than those derived from partial-correlation-based networks for both short-term scans (WOGR-PEAR>WOGR-PAR: p = 0.04) and long-term scans (WGR-PEAR>WGR-PAR: p = 0.003; WOGR-PEAR>WOGR-PAR: p = 0.016). Global signal regression produced less reliable results for short-term scans (WOGR-PEAR>WGR-PEAR: p = 0.019) but no significant differences for long-term scans (p = 0.46).

**Figure 6:**
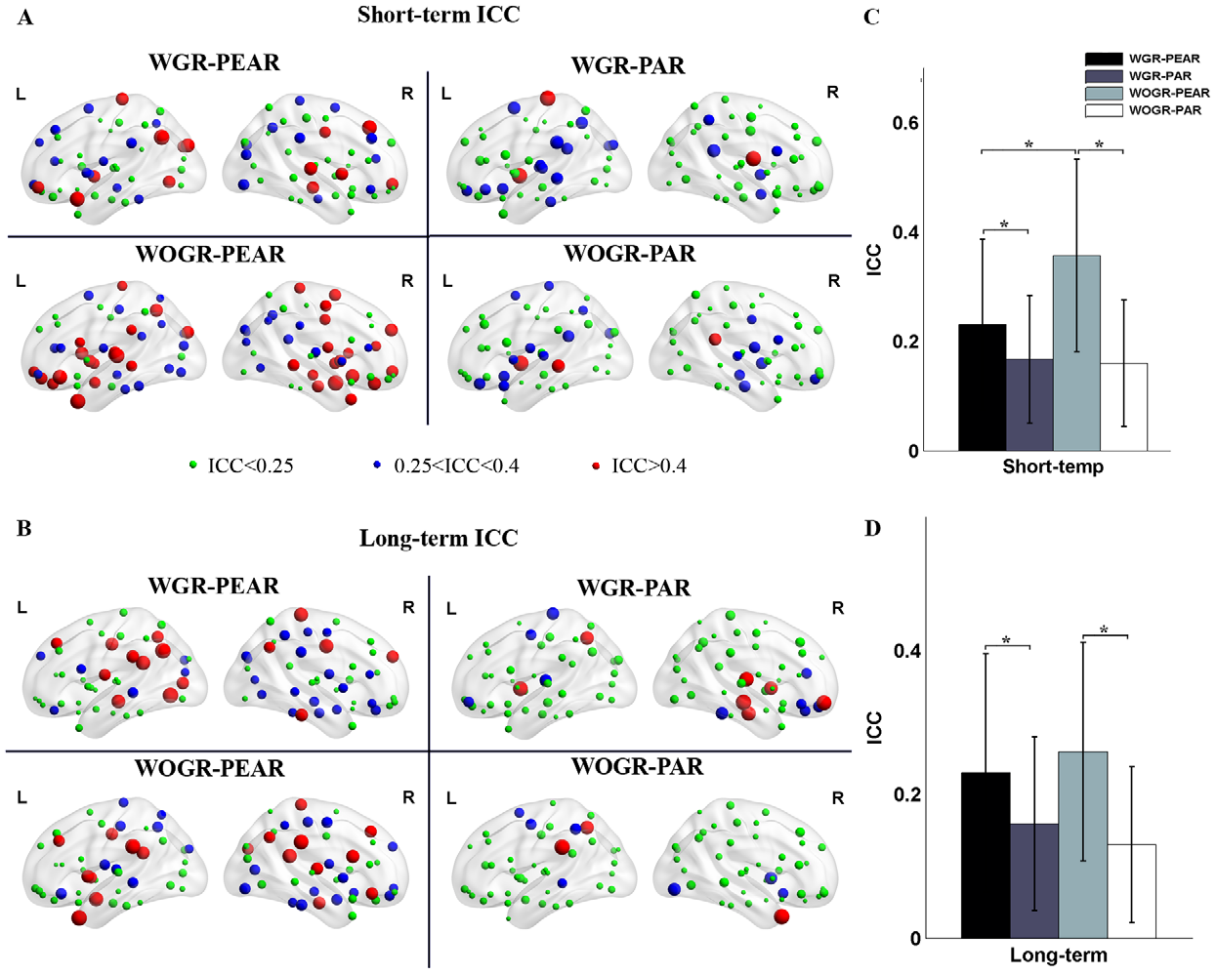
TRT reliability of nodal degree for Pearson's-correlation and partial-correlation-based networks with and without global signal regression. Nodal TRT reliability values were projected onto MNI brain surface using the BrainNet viewer (http://www.nitrc.org/projects/bnv/) for (A) short-term scans and (B) long-term scans in WGR-PEAR networks, WGR-PAR networks, WOGR-PEAR networks and WOGR-PAR networks. Significant differences were found in TRT reliability of nodal degree driven by correlation metrics (Pearson's correlation/partial correlation) and global signal regression (with/without) for (C) short-term and (D) long-term scans. Nodal degree in WOGR-PEAR networks showed the highest ICC values. The asterisk indicates p<0.05. L, left hemisphere; R, right hemisphere.

We next evaluated the reliability of nodal degree in brain networks under the four different processing schemes, and the reliability of nodal degree was rendered onto brain surfaces (Fig. 6A, B). Our results showed that in Pearson's-correlation-based networks (WGR-PEAR and WOGR-PEAR networks), brain regions with fair reliability were predominately located in the association and limbic/paralimbic cortexes (Mesulam 1998). The association cortex regions include the left ANG, right paracentral lobe (PCL), right precunues (PCUN), bilateral superior frontal gyrus (dorsolateral) (SFGdor) and right ITG. The limbic/paralimbic regions include the bilateral hippocampus (HIP), bilateral MCG and left posterior cingulate gyrus (PCG). In addition, one primary cortex region of the left CAL was found to be fairly reliable. In partial-correlation-based networks, certain limbic/paralimbic and subcortical cortex regions exhibited fair reliability including the left PCUN, right HIP, right parahippocampal gyrus (PHG) and bilateral PUT. Statistical analysis of nodal reliability revealed that the TRT reliability of nodal degree was modulated by the two processing factors: Pearson's-correlation-based networks were more reliable than partial-correlation-based networks for both short-term scans (WGR-PEAR>WGR-PAR: p = 7.6e^−7^; WOGR-PEAR>WOGR-PAR: p = 1.2e^−14^) and long-term scans (WGR-PEAR>WGR-PAR: p = 3.6e^−8^; WOGR-PEAR>WOGR-PAR: p = 1.1e^−10^) (Fig. 6C, D). Networks without global signal regression were more reliable for both short-term scans (WOGR-PEAR>WGR-PEAR: p = 2.2e^−7^) and long-term scans (WOGR-PEAR>WGR-PEAR: p = 0.03) (Fig. 6C, D). These results suggest that WOGR-PEAR networks exhibit the most reliable topological properties for both short-term and/or long-term scans.

### Frequency bands: dependence and reliability

To assess the influence of frequency band selection on network topology, we next divided the widely used frequency interval (0.01–0.1 Hz) into two sub-bands: slow-5 (0.01–0.027 Hz) and slow-4 (0.027–0.073 Hz) and re-performed the network analysis in these two bands in the case of WOGR-PEAR, given that the WOGR-PEAR networks exhibit the most reliable global and nodal topological architecture as shown above.

#### Effects on network topology

**Figure 7:**
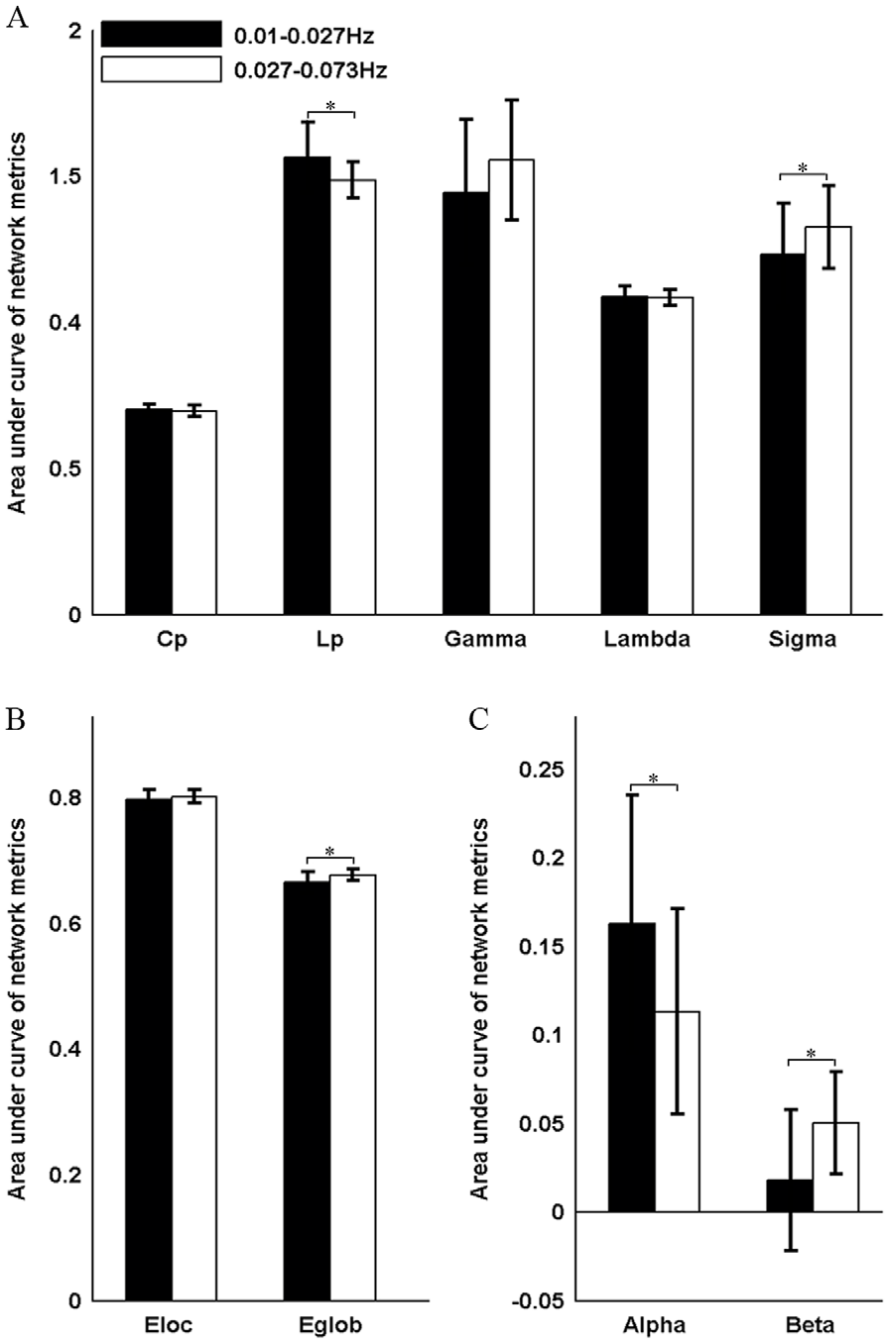
Frequency dependent differences in global network properties of functional brain networks. Bars show the differences in the areas under curves (AUC) of (A) small-world parameters (Cp, Lp, γ, λ and σ), (B) network efficiency (Local efficiency and Global efficiency), (C) assortativity coefficient (α) and hierarchy coefficient (β). Error bars correspond to standard deviation of the mean across participants. The asterisk indicates p<0.05.

**Figure 8:**
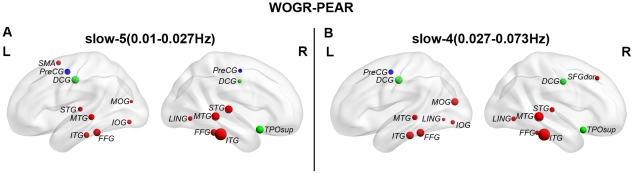
Functional hubs derived from networks in different frequency bands. Regions with degree>the mean+standard deviation are considered to be hubs. Node colors were coded according to their membership of classical cortex classifications: association cortex (red), limbic cortex (purple), paralimbic cortex (green), subcortical regions (light blue) and primary cortex regions (dark blue). (A) slow-5 (0.01–0.027 Hz). (B) slow-4 (0.027–0.073 Hz).

There was no significant difference in the clustering coefficients or local efficiency of brain graphs driven by the different frequency bands. However, brain networks constructed in the 0.027–0.073 Hz frequency band were found to be more globally efficient than those in 0.01–0.027 Hz as indicated by shorter Lp (p = 0.0007) and greater global efficiency (p = 0.001) (Fig. 7). Additionally, our results revealed that brain networks in slow-4 are less assortative (p = 0.002) but more hierarchical (p = 3.79e^−9^) than those in slow-5 (Fig. 7). These results indicate that the selection of different frequency bands can have a significant influence on the global topological properties of functional brain networks. Fig. 8 shows the hubs in WOGR-PEAR networks in the two different frequency bands. Similar spatial patterns of network hubs were observed in the two frequency intervals mainly in association cortex regions (e.g., ITG, MTG, fusiform gyrus [FFG] and SFGdor) and paralimbic/limbic cortex regions (e.g., MCG, TPOsup and TPOmid) with a high correlation coefficient (r = 0.85, p = 0.00).

#### TRT reliability of network metrics

**Figure 9:**
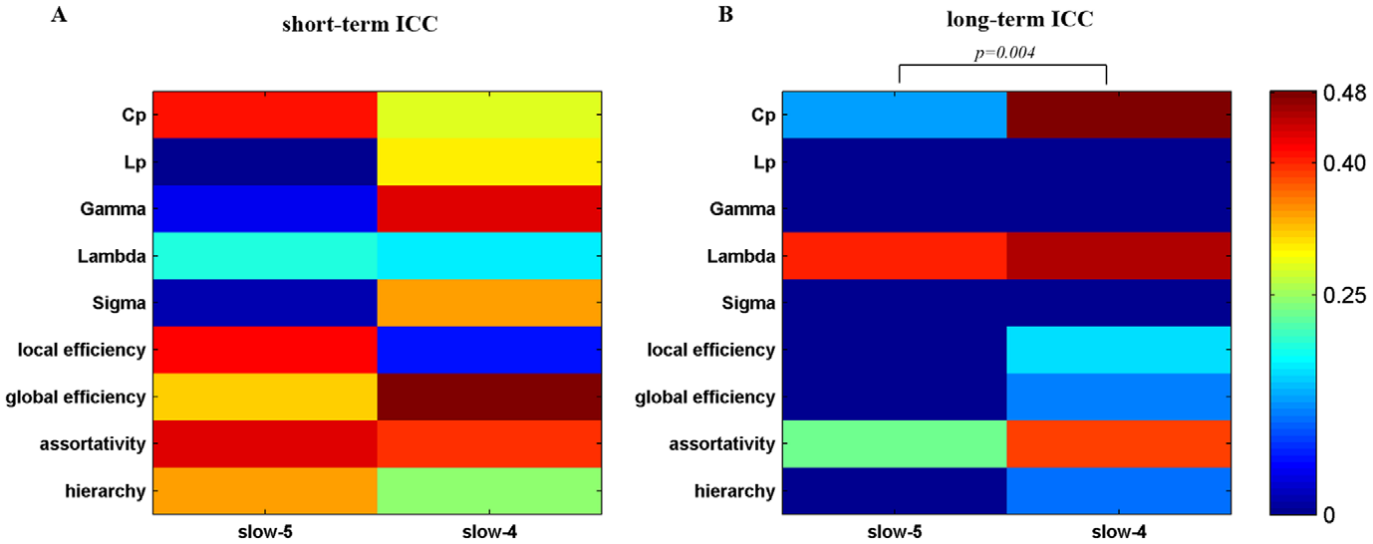
TRT reliability of global topological properties for networks in different frequency bands. The reliability was estimated using areas under curves (AUC) of each metric. Statistical analysis revealed significant differences in (A) short-term and/or (B) long-term TRT reliability driven by different frequency bands.

**Figure 10:**
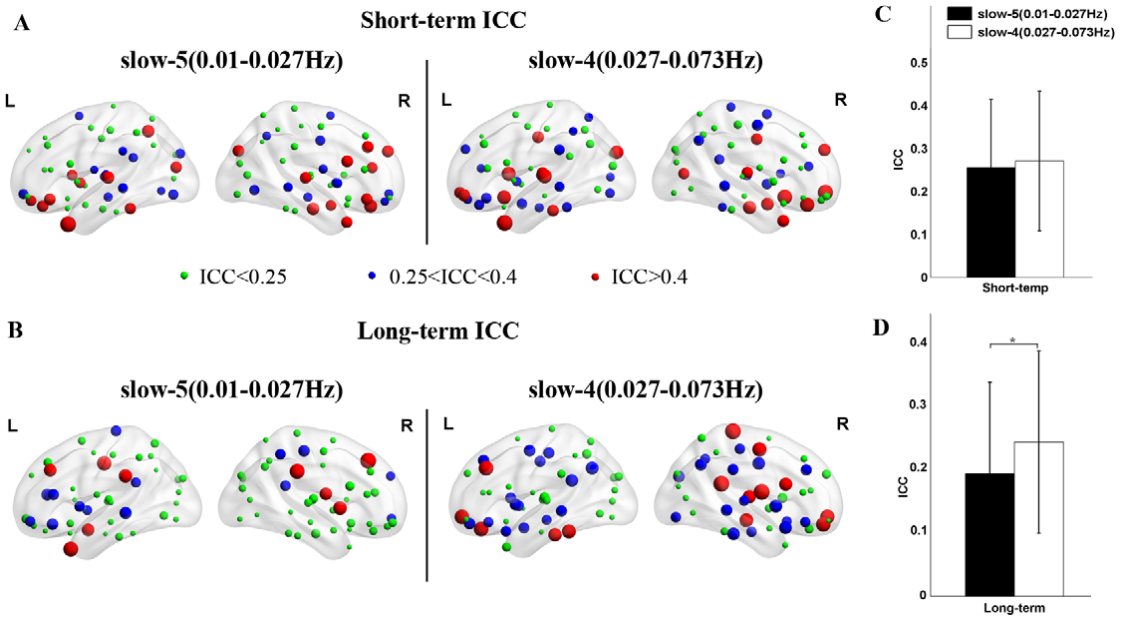
TRT reliability of nodal degree for brain networks in different frequency bands. TRT reliability values of nodal degree were projected onto MNI brain surface using the BrainNet viewer (http://www.nitrc.org/projects/bnv/) for (A) short-term scans and (B) long-term scans in slow-5 and slow-4. Significant differences were found in TRT reliability of nodal degree driven by different frequency bands for (C) short-term and (D) long-term scans. Nodal degree in brain networks in slow-4 band showed higher ICC values. The asterisk indicates p<0.05. L, left hemisphere; R, right hemisphere.

The brain networks in both frequency bands showed overall low reliability (Fig. 9). The TRT reliability of global brain network metrics appeared to be greater in slow-4 (0.027–0.073 Hz) than in slow-5 (0.01–0.027 Hz) by visual inspection. Subsequent statistical comparisons revealed that the TRT reliability of global network metrics was modulated by the frequency band with higher reliability observed for networks in slow-4 for long-term scans (p = 0.004) but not for short-term scans (p = 0.65). Nodal degree in the slow-5 and slow-4 brain networks exhibited similar short-term reliability with fair reliability regions mostly located in association and limbic/paralimbic cortex regions such as the right medial superior frontal gyrus (SFGmed), right medial orbitofrontal cortex (ORBmed), left ITG and bilateral MCG (Fig. 10A). Regions with fair long-term nodal degree reliability were also located in association and limbic/paralimbic cortex regions. However, networks in the slow-4 band displayed more fair reliability regions than slow-5 band networks including the left FFG, left ITG, bilateral ORBmed, right PCL, right PCG and right HIP (Fig. 10B). Statistical comparisons revealed that the nodal degree of brain networks in slow-4 showed greater reliability than those in slow-5 for long-term scans (p = 0.0018) but not for short-term scans (p = 0.26) (Fig. 10C, D). Note that for short-term scans, even not significant, slow-4 demonstrates greater ICC values than slow-5 on visual inspection, indicating a trend of frequency-dependent differences in reliability for short-term scans. The strengthened differences in reliability between slow-4 and slow-5 for long-term scans could be a result of decreased inter-scan reliability in slow-5 band. We performed statistical tests between inter-scan and intra-scan ICCs in the two slow bands, and confirmed that reliability is significant lower for long-term scans than short-term scans in slow-5 band (p = 0.004) but not in slow-4 band (p = 0.18). These results suggest that brain networks constructed in the slow-4 band are more reliable than those in slow-5, which may reflect the fact that different frequency bands could be associated with different physiological functions (Buzsaki and Draguhn, 2004). Nevertheless, further work is needed to verify this finding and investigate the specific brain function in different frequency bands.

## Discussion

In this study, we investigated the influences of different correlation metrics and preprocessing steps (the application of global signal regression and the selection of a frequency band) on the topological properties of functional brain networks obtained using R-fMRI. Our results showed that both the global (C_p_, L_p_, γ , λ, E_loc_, E_glob_, α and β) and regional (nodal degree) topological properties of brain networks depend heavily on the correlation method (Pearson's correlation/partial correlation) used and the application of global signal removal. TRT analysis showed that Pearson's-correlation-based brain networks with global signals conserved (WOGR-PEAR) had the most reliable topological properties. We further found that there were significant frequency-related differences in topological properties in WOGR-PEAR networks. Brain networks in 0.027–0.073 Hz exhibited greater reliability than to those in 0.01–0.027 Hz. These results provide quantitative evidence regarding the influence of correlation metrics and specific preprocessing choices on both the global and nodal topological properties of functional brain networks. This study also has important implications on how to choose reliable analytical schemes and for the application of brain network studies under healthy and pathological conditions.

### Efficient small-world functional brain networks

We found robust small-world properties in all functional brain networks regardless of the correlation metric or global signal regression (Fig. 2), which is in accordance with previous network studies [7], [17], [20]. We also found that functional brain networks constructed in different frequency bands all possess small-world organization (Fig. S1, S2, S3, S4), which has also been shown in previous studies [7], [39]. These results indicate that small-world architecture is well-suited and prevalent for functional brain networks with respect to densely connected clusters bridged by short paths, which may maximize the efficiency of information processing at a relatively low wiring cost [55].

In addition, the functional brain networks appeared to be assortative and hierarchical, irrespective of the correlation metric, global signal presence or frequency band. Assortativity is the tendency for a high degree hub to connect preferentially to other hubs, and this organization is most common in social networks. It has been shown that many networks can break down (become disconnected between pairs of nodes) by removing just a few of the hubs. However, Newman [56] revealed that assortative networks are robust against targeted removal of high-degree nodes, probably because the targeted hubs tend to be united together and removing them is somewhat redundant. Our results show that functional brain networks are assortative, which is consistent with previous studies [57], [58] and may suggest that functional brain systems are resilient when facing pathological attacks. Hierarchical networks are composed of numerous small, integrated clusters that are assembled into larger, less integrated groups. This self-similar nesting structure can be captured by the scaling relationship between the clustering coefficient and the network node degree. Hubs in hierarchical networks prefer to connect with nodes that have a small chance of linking to each other, which enables them to play the important role of bridging the small clusters into a single, integrated network [59]. Our observation that functional networks are hierarchical corresponds well with previous studies [39], [44], [45].

### Effects of correlation metrics on brain network topology

We found significant correlation metric-dependent discrepancies in the global topological attributes of functional brain networks, which is in accordance with a recent EEG study [60]. Our results strongly indicate that most global topological parameters are sensitive to the correlation metric used. The observed differences should be attributable to the inherent properties of each metric. Pearson's correlation measures the interdependence between two time series. However, the correlation value may result from indirect relationship caused by common sources. Partial correlation parcels out common driver components and measures the direct temporal relationship between brain regions [23]. Including indirect connections in a network may have an influence on topological parameters. In addition, the methodological distinction may also result in differences in the strength and distribution of functional connectivity, thus accounting for the observed between-group differences in certain network attributes.

Regional network analysis revealed a distinct spatial pattern of hubs between Pearson's-correlation-based networks and partial-correlation-based networks. Hubs in the former were predominately composed of association cortical regions, which were in accordance with both functional [7], [10] and structural [61] network studies using Pearson's correlation, whereas hubs in the latter also appeared in primary cortex, paralimbic/limbic cortex and subcortical regions. There were very few studies have used partial correlation for hub identification. A structural network study has found hub regions mostly distributed in association cortex regions, and only 2 paralimbic cortex regions and 1 primary motor region were identified as hubs [62]. However, these results are hardly comparable with the present study because of different imaging modalities, different parcellation schemes (node definition) and different nodal metrics used for hub identification. The discrepancy in the spatial pattern of hub regions between Pearson's- and partial-correlation-based networks is not completely understood. A possible explanation could be that association cortex regions integrate multiple perceptive and cognitive functional systems [63] and thus play an important role in functional brain networks regardless of the connectivity metric used. Paralimbic/limbic and subcortical regions with extensive direct projections have been shown to mediate the homeostatic and autonomic aspects of the internal milieu [63]. These relatively isolated functions may account for their non-hub status in Pearson's correlated brain networks, whereas their extensive direct connections make them hubs in partial correlated networks because partial correlation counts only the direct interactions between brain regions.

TRT analysis showed that the reliability of global and nodal network metrics is modulated by the correlation metric used. Pearson's-correlation-based brain networks tended to have higher TRT reliability than partial-correlation-based networks. Possible reasons for this observation are due to the emergence of negative connectivity related to the partial correlation method. Previous studies have shown that negative correlations reduce the TRT reliability of functional connectivity [48] and network metrics [44]. Another reason could stem from validity issues surrounding the partial correlation method. Although partial correlation is prominent for its ability to cut indirect edges, it raises the opposite problem of losing real connections, which could lead to false-negative errors. A recent study demonstrated that partial correlation performs poorly at detecting connections in networks with a large number of node [25]. Together with this evidence, our results suggest that Pearson's correlation is more valid and reliable, whereas partial correlation should be treated with caution for resting-state brain network studies.

### Effects of the global signal on brain network topology

We found significant global signal-related differences in the global network parameters of Pearson's-correlation-based networks, which may be associated with the effects of global signal correction on functional correlativity. These effects include the improved specificity of positive correlations and the emergence of negative correlations [27], [28], [30]. Although the exact biological basis of the global signal and the effect of its removal are not fully understood [30], [31], our results indicate that global signal correction may have a broad influence on the global topological properties of Pearson's-correlation-based networks.

The spatial pattern of hubs was broadly consistent between the global signal-removed and conserved brain networks. Similar results have been previously observed in the modular organization of functional brain networks [10]. This relatively stable regional topology indicates that the global signal might not significantly influence the regional architecture of functional brain networks.

TRT analysis revealed that brain networks without global signal removal outperform those with global signal removal with respect to the TRT reliability of their global topological properties, suggesting that the global signal should not be removed in Pearson's-correlation-based brain networks in terms of its TRT reliability.

### Effects of frequency band on brain network topology

It has been found that the neuroelectric oscillations in different frequency bands progress linearly on the logarithmic scale [35] and are specifically associated with a variety of cognitive functions [64]. In the present study, on the basis of the approach of Penttonen and Buzsaki, we focused on the widely adopted frequency band (0.01–0.1 Hz) of spontaneous BOLD fluctuations and divided it into a slow-5 band (0.01–0.027 Hz) and a slow-4 band (0.027–0.073 Hz). By performing network analysis on both bands, we found that the small-world metrics (including global efficiency and 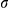) were significantly higher in the slow-4 band than in the slow-5 band, which is consistent with previous studies [7], [39]. An interesting observation was that frequency has influence on global efficiency but not network hub. This could be a result with regard to the different roles of network hubs. Although networks in slow-5 and slow-4 bands have similar hub distribution, these hub regions could play different roles in different frequency bands. For example, a region could be a provincial hub in one frequency band but a connector hub in another frequency band. Provincial hubs are nodes that are highly connected by connections that are mostly contained within a single modular of the network, on the contrary, connections of connector hubs run mostly between two or more modules [65], [66]. Such discrepancy in hub roles could result in differences in global efficiency of the networks between slow-5 and slow-4 band. Future studies have to validate this and investigate the detailed hub roles in different frequency bands. Furthermore, TRT reliability analysis revealed the slow-4 band was more reliable for both global and regional network metrics than the slow-5 band. These results are in accordance with previous findings that showed that the slow-4 band has higher TRT reliability and more reliable BOLD fluctuation amplitude voxels than the slow-5 band [48]. Moreover, in fMRI-based studies, ADHD children have presented more diagnostic information in the slow-4 band than in other bands [47]. Patients with schizophrenia have been found to show widespread LFO amplitude abnormalities in the slow-4 frequency band [67]. Together with these findings in both normal and diseased populations, our results indicate that the topological properties of functional brain networks are dependent on the frequency interval used and are more reliable in the slow-4 band.

### Methodological considerations

Choosing the ‘best’ processing step during network modeling is a very difficult task given the lack of a golden standard [12], [68]. In this study, we performed a systematic analysis of factors influencing brain networks and their TRT reliability. In addition, using balloon models to simulate functional brain networks is also likely to offer some informative guidance. By applying different processing choices to simulated networks with known topological properties, the processing pipeline resulting in the most accurate network model can be determined. Recently, Smith et al. [25] performed a comprehensive analysis with simulated fMRI data to validate multiple connectivity metrics, including the Pearson's and partial correlation metrics discussed in our present paper. They showed that these two connectivity metrics are very successful at estimating functional connectivity in general but that Pearson's correlation performs better when the network node number dramatically increases. This finding is confirmed by our TRT results, which show that the topological properties of brain networks constructed using Pearson's correlation are more reliable than those using partial correlation. It would be interesting to conduct simulated studies to validate different network estimation methods in the context of graph analysis, but this is beyond the scope of this paper.

Another consideration is the interaction between network node and edge definitions. As mentioned before, how to define nodes and edges are two key questions in network construction, and they are of importance in network studies. Network nodes in fMRI data can be defined according to a prior parcellated template or by sampling clusters of equal numbers of voxels. It has been shown that network topological properties can vary across different anatomical templates [17] and different spatial nodal sizes [18], but no absolutely standard approach to define network nodes currently exists. The analysis of edge definition in our present study is limited to AAL template-based brain networks, and caution should be exercised when applying the current results to networks constructed using other node definitions. Future studies will be required to clarify the interactions between node and edge definitions.

Moreover, the two datasets used here were acquired under different eyes conditions, eyes closed for dataset 1 and eyes open for dataset 2. Recent studies have shown that regional activities (e.g. ALFF, amplitude of low frequency fluctuations) and functional connectivity within the default and attention networks were significantly decreased for eyes closed scans comparing with eyes opened (or fixation) scans [69], [70]. However, whether these differences would result in topological alterations of brain network remains unclear. Future studies should be conducted to clarify this issue by collecting eyes closed and eyes opened R-fMRI data on the same subjects. In addition, the present study only investigated the influences of the two most frequently used correlation metrics, Pearson's correlation and partial correlation. Many other connectivity metrics exist for functional network analysis, such as mutual information and synchronization likelihood. Further work is needed to clarify the influences of other metrics on brain network architecture. Finally, given that the topology of global brain network and/or certain brain regions would be disrupted in diseased and aging people [3], [11], [12], [13], and thus render themselves more vulnerable or less reliable than normal controls, it would be interesting to explore possible specificities of such population with respect to specific correlation metrics or preprocessing steps, which may have important implications in the application of network analysis to the healthy and diseased brain.

### Conclusion

The current work represents a systematic and quantitative evaluation of the effects of different processing choices on the architecture and TRT reliability of R-fMRI brain networks. Our results indicate that such brain networks have robust small-world configurations regardless of the correlation metric or preprocessing strategy (global signal regression and frequency interval) employed, but significant differences exist in both their global and regional topological parameters. These results suggest that comparisons of network studies with different processing strategies should be viewed with caution. TRT analysis showed that the reliability of network parameters was modulated by all three processing factors. In particular, topological properties were at their most reliable in the WOGR-PEAR networks. Furthermore, the TRT reliability of topological parameters was higher in the slow-4 band (0.027–0.073 Hz) than in the slow-5 band (0.01–0.027 Hz). Our results shed light on the processing strategies of functional brain networks acquired from resting-state fMRI. Our findings also provide a foundation for continued examination of network properties in typical and atypical populations.

## Supporting Information

Figure S1 Global topological properties in WGR-PEAR brain networks at different frequency bands. Plots show the changes in small-world parameters (Cp, Lp, γ, λ and σ), network efficiency (Local efficiency and Global efficiency), assortativity coefficient (α) and hierarchy coefficient (β) in functional brain networks at two different frequency bands (slow-5: 0.01–0.027 Hz, slow-4: 0.027–0.073 Hz) in case of WGR-PEAR as a function of sparsity thresholds. Local and global efficiency of random and regular networks with the same number of nodes and edges as the real networks were shown in gray lines in the network efficiency plots.
(TIF)

Figure S2 Global topological properties in WGR-PAR brain networks at different frequency bands. Plots show the changes in small-world parameters (Cp, Lp, γ, λ and σ), network efficiency (Local efficiency and Global efficiency), assortativity coefficient (α) and hierarchy coefficient (β) in functional brain networks at two different frequency bands (slow-5: 0.01–0.027 Hz, slow-4: 0.027–0.073 Hz) in case of WGR-PAR as a function of sparsity thresholds. Local and global efficiency of random and regular networks with the same number of nodes and edges as the real networks were shown in gray lines in the network efficiency plots.
(TIF)

Figure S3 Global topological properties in WOGR-PEAR brain networks at different frequency bands. Plots show the changes in small-world parameters (Cp, Lp, γ, λ and σ), network efficiency (Local efficiency and Global efficiency), assortativity coefficient (α) and hierarchy coefficient (β) in functional brain networks at two different frequency bands (slow-5: 0.01–0.027 Hz, slow-4: 0.027–0.073 Hz) in case of WOGR-PEAR as a function of sparsity thresholds. Local and global efficiency of random and regular networks with the same number of nodes and edges as the real networks were shown in gray lines in the network efficiency plots.
(TIF)

Figure S4 Global topological properties in WOGR-PAR brain networks at different frequency bands. Plots show the changes in small-world parameters (Cp, Lp, γ, λ and σ), network efficiency (Local efficiency and Global efficiency), assortativity coefficient (α) and hierarchy coefficient (β) in functional brain networks at two different frequency bands (slow-5: 0.01–0.027 Hz, slow-4: 0.027–0.073 Hz) in case of WOGR-PAR as a function of sparsity thresholds. Local and global efficiency of random and regular networks with the same number of nodes and edges as the real networks were shown in gray lines in the network efficiency plots.
(TIF)

Figure S5 Correlation metrics and global signal dependent differences in global network properties in weighted networks. Bars show the differences in the areas under curves (AUC) of (A) small-world parameters (Cp, Lp, γ, λ and σ), (B) network efficiency (Local efficiency and Global efficiency) and (C) assortativity coefficient (α) and hierarchy coefficient (β). Error bars correspond to standard deviation of the mean across participants. The asterisk indicates p<0.05.
(TIF)

Figure S6 Functional hubs derived from weighted networks using different correlation metrics and global signal strategies. (A) WGR-PEAR, (B) WOGR-PEAR, (C) WGR-PAR and (D) WOGR-PAR. Regions with degree>the mean+standard deviation were considered hubs. Node colors were coded according to their membership of classical cortex classifications: association cortex (red), limbic cortex (purple), paralimbic cortex (green), subcortical regions (light blue) and primary cortex regions (dark blue).
(TIF)

Figure S7 TRT reliability of global topological properties for Pearson's-correlation and partial-correlation-based weighted networks with and without global signal regression. The reliability was estimated using areas under curves (AUC) of each metric. Statistical analysis revealed significant differences in (A) short-term and/or (B) long-term TRT reliability driven by correlation metrics and/or global signal regression.
(TIF)

Figure S8 TRT reliability of nodal degree for Pearson's-correlation and partial-correlation-based weighted networks with and without global signal regression. Nodal TRT reliability values were projected onto MNI brain surface using the BrainNet viewer (http://www.nitrc.org/projects/bnv/) for (A) short-term scans and (B) long-term scans in WGR-PEAR networks, WGR-PAR networks, WOGR-PEAR networks and WOGR-PAR networks. Significant differences were found in TRT reliability of nodal degree driven by correlation metrics (Pearson's correlation/partial correlation) and global signal regression (with/without) for (C) short-term and (D) long-term scans. Nodal degree in WOGR-PEAR networks showed the highest ICC values. The asterisk indicates p<0.05. L, left hemisphere; R, right hemisphere.
(TIF)

Figure S9 Frequency dependent differences in global network properties of weighted functional brain networks. Bars show the differences in the areas under curves (AUC) of (A) small-world parameters (Cp, Lp, γ, λ and σ), (B) network efficiency (Local efficiency and Global efficiency), (C) assortativity coefficient (α) and hierarchy coefficient (β). Error bars correspond to standard deviation of the mean across participants. The asterisk indicates p<0.05.
(TIF)

Figure S10 Functional hubs derived from weighted networks in different frequency bands. Regions with degree>the mean+standard deviation are considered to be hubs. Node colors were coded according to their membership of classical cortex classifications: association cortex (red), limbic cortex (purple), paralimbic cortex (green), subcortical regions (light blue) and primary cortex regions (dark blue). (A) slow-5 (0.01–0.027 Hz). (B) slow-4 (0.027–0.073 Hz).
(TIF)

Figure S11 TRT reliability of global topological properties for weighted networks in different frequency bands. The reliability was estimated using areas under curves (AUC) of each metric. Statistical analysis revealed significant differences in (A) short-term and/or (B) long-term TRT reliability driven by different frequency bands.
(TIF)

Figure S12 TRT reliability of nodal degree for weighted brain networks in different frequency bands. TRT reliability values of nodal degree were projected onto MNI brain surface using the BrainNet viewer (http://www.nitrc.org/projects/bnv/) for (A) short-term scans and (B) long-term scans in slow-5 and slow-4. Significant differences were found in TRT reliability of nodal degree driven by different frequency bands for (C) short-term and (D) long-term scans. Nodal degree in brain networks in slow-4 band showed higher ICC values. The asterisk indicates p<0.05. L, left hemisphere; R, right hemisphere.
(TIF)

Table S1 Regions of interest from AAL atlas. The regions are listed in terms of a prior template of Anatomical Automatic Labeling atlas (Tzourio-Mazoyer et al., 2002).
(DOC)

Text S1 (DOC)
