# Supplementary figures and images for: Effects of Different Correlation Metrics and Preprocessing Factors on Small-World Brain Functional Networks: A Resting-State Functional MRI Study
Source: PLoS One. 2012 Mar 6;7(3):e32766. doi: 10.1371/journal.pone.0032766 (PMC3295769; doi:10.1371/journal.pone.0032766)

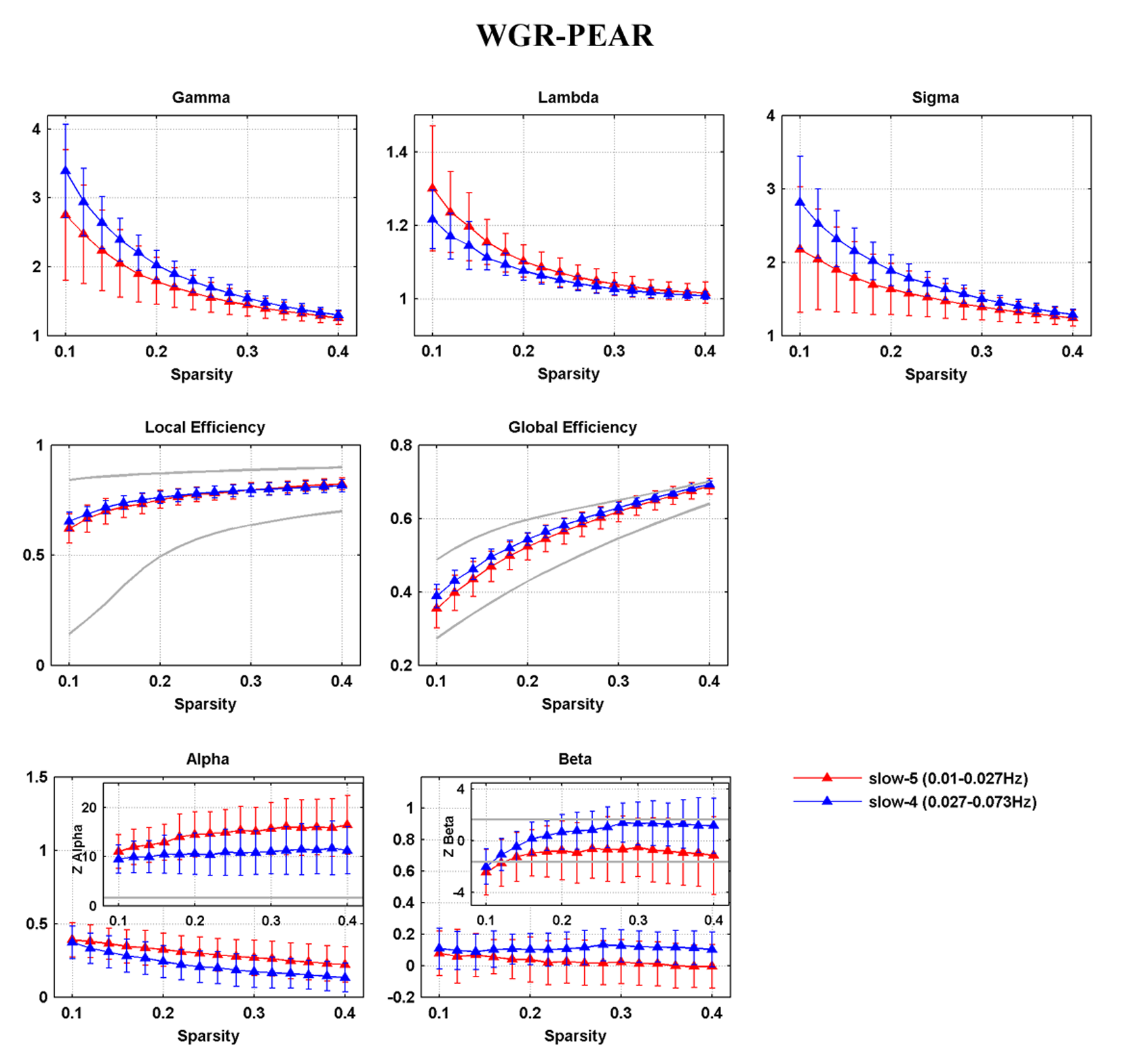

Supplement: Figure S1 — Global topological properties in WGR-PEAR brain networks at different frequency bands. Plots show the changes in small-world parameters (Cp, Lp, γ, λ and σ), network efficiency (Local efficiency and Global efficiency), assortativity coefficient (α) and hierarchy coefficient (β) in functional brain networks at two different frequency bands (slow-5: 0.01–0.027 Hz, slow-4: 0.027–0.073 Hz) in case of WGR-PEAR as a function of sparsity thresholds. Local and global efficiency of random and regular networks with the same number of nodes and edges as the real networks were shown in gray lines in the network efficiency plots. (TIF) [file pone.0032766.s001.tif]

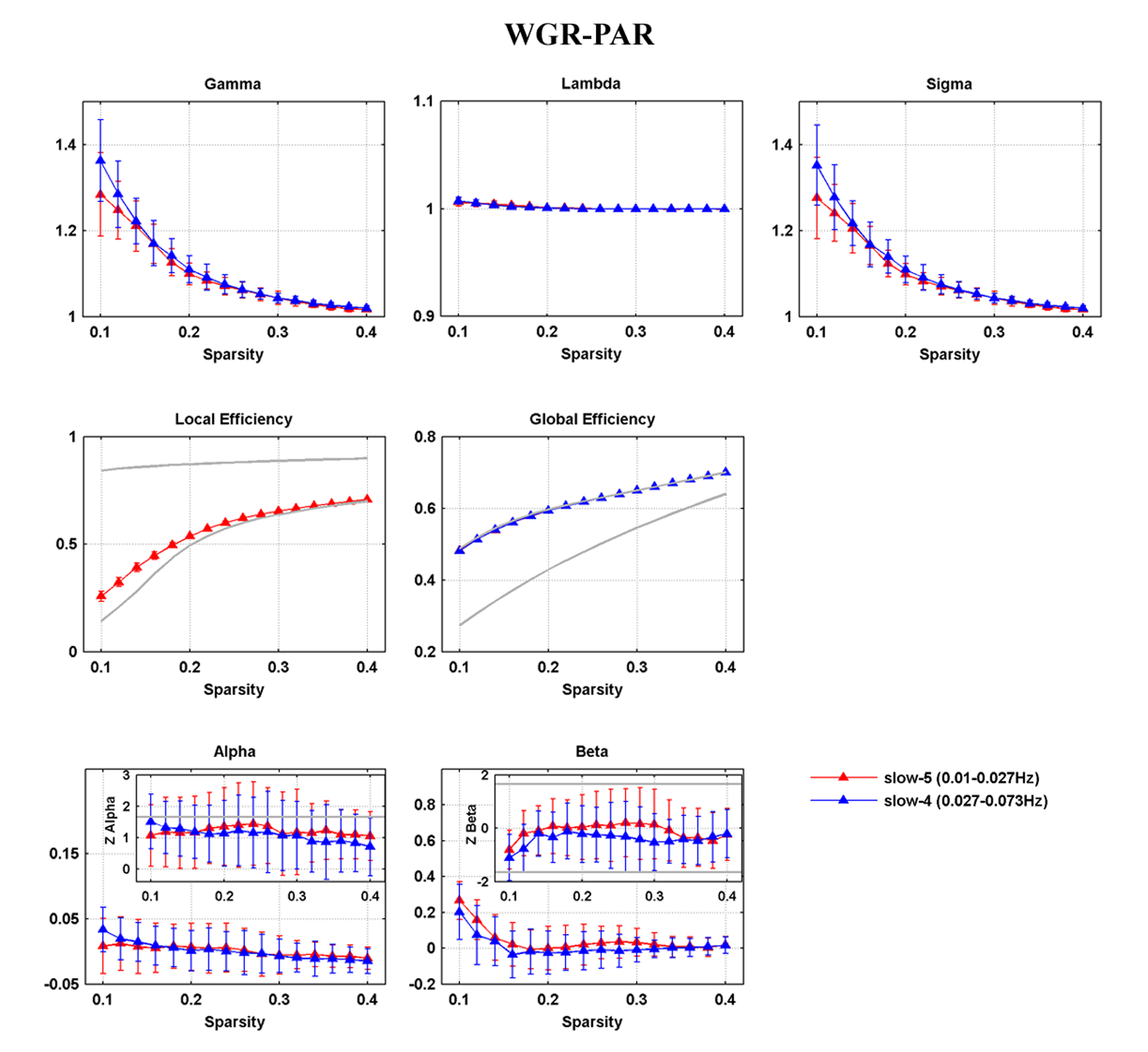

Supplement: Figure S2 — Global topological properties in WGR-PAR brain networks at different frequency bands. Plots show the changes in small-world parameters (Cp, Lp, γ, λ and σ), network efficiency (Local efficiency and Global efficiency), assortativity coefficient (α) and hierarchy coefficient (β) in functional brain networks at two different frequency bands (slow-5: 0.01–0.027 Hz, slow-4: 0.027–0.073 Hz) in case of WGR-PAR as a function of sparsity thresholds. Local and global efficiency of random and regular networks with the same number of nodes and edges as the real networks were shown in gray lines in the network efficiency plots. (TIF) [file pone.0032766.s002.tif]

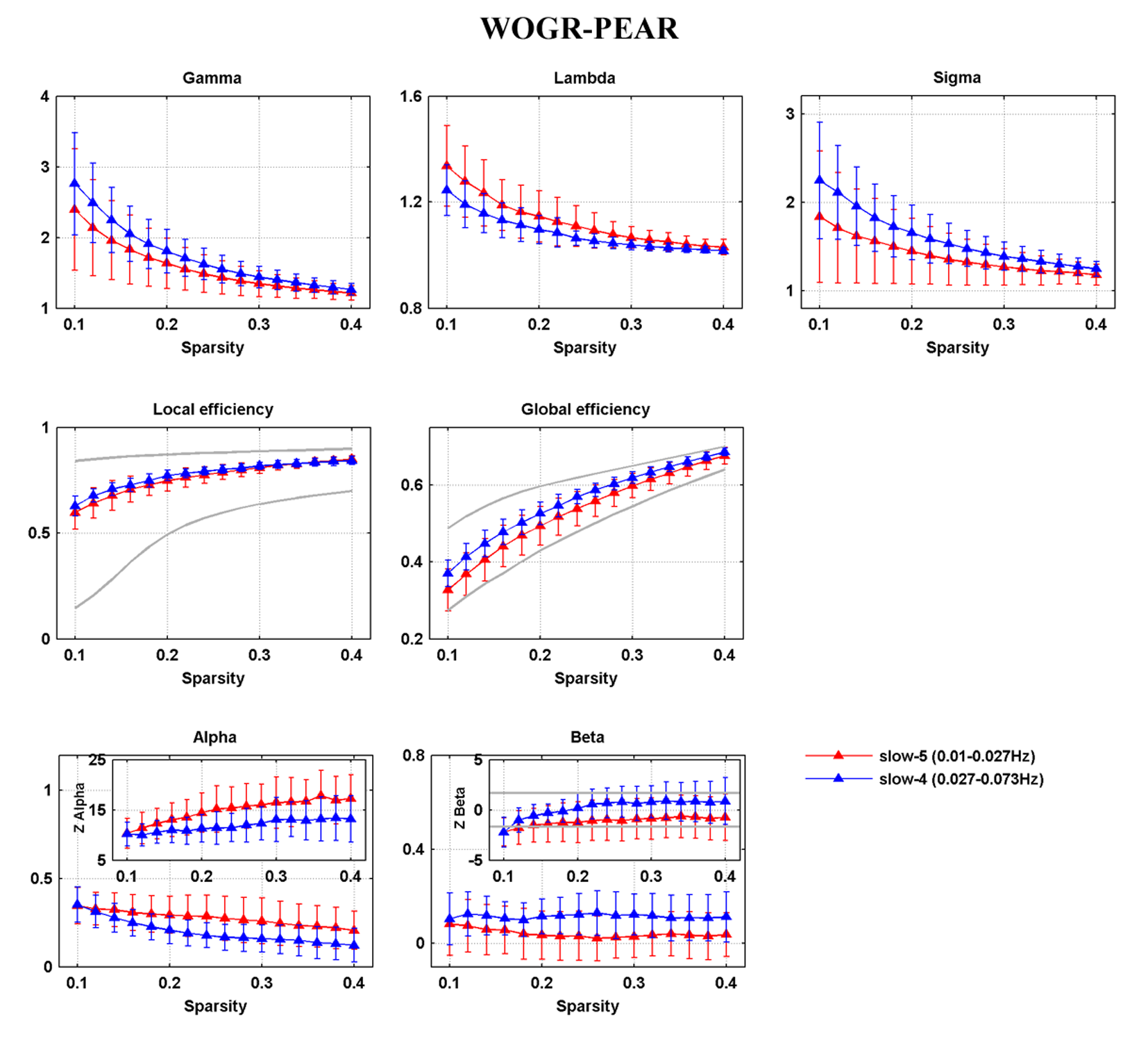

Supplement: Figure S3 — Global topological properties in WOGR-PEAR brain networks at different frequency bands. Plots show the changes in small-world parameters (Cp, Lp, γ, λ and σ), network efficiency (Local efficiency and Global efficiency), assortativity coefficient (α) and hierarchy coefficient (β) in functional brain networks at two different frequency bands (slow-5: 0.01–0.027 Hz, slow-4: 0.027–0.073 Hz) in case of WOGR-PEAR as a function of sparsity thresholds. Local and global efficiency of random and regular networks with the same number of nodes and edges as the real networks were shown in gray lines in the network efficiency plots. (TIF) [file pone.0032766.s003.tif]

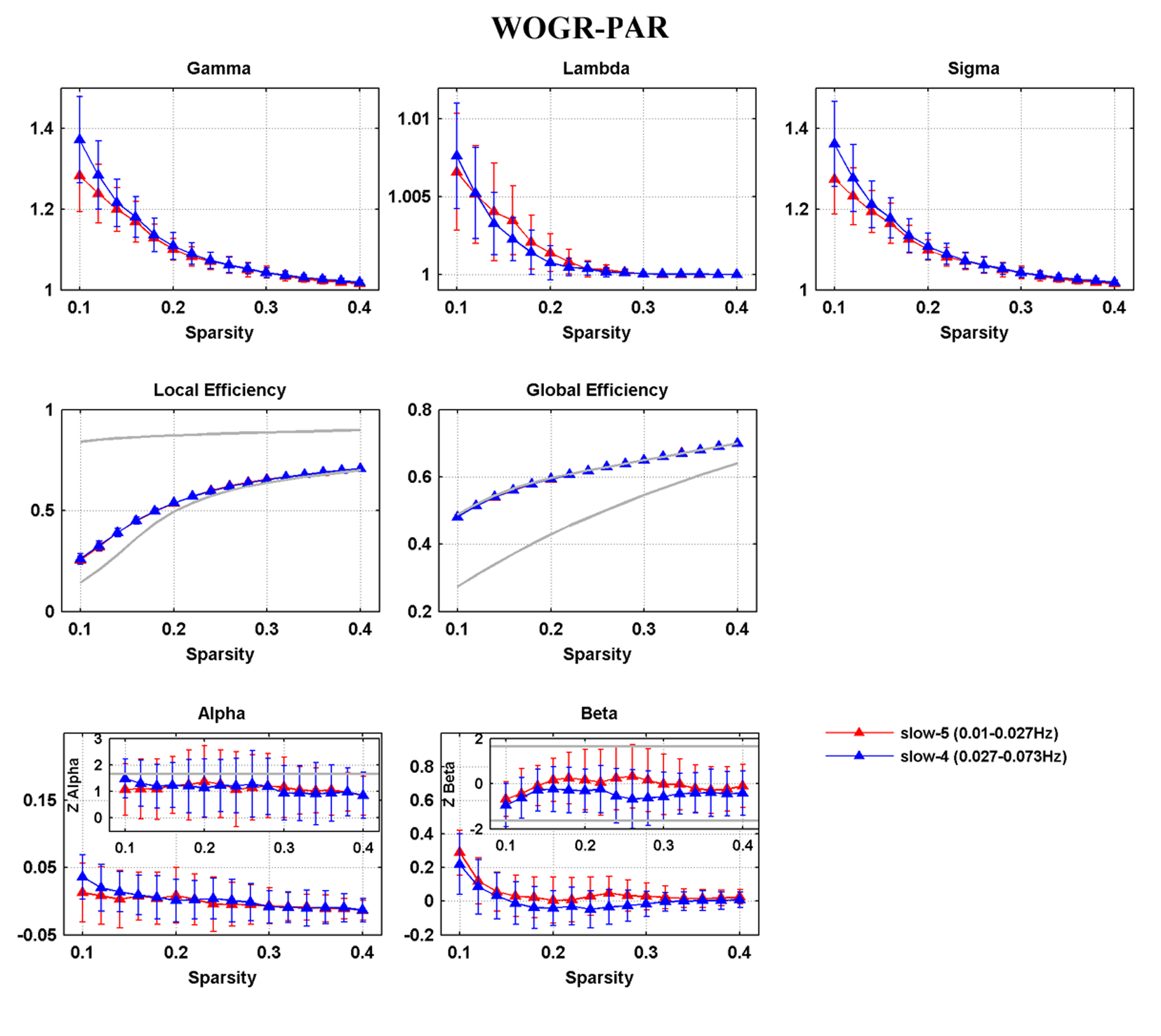

Supplement: Figure S4 — Global topological properties in WOGR-PAR brain networks at different frequency bands. Plots show the changes in small-world parameters (Cp, Lp, γ, λ and σ), network efficiency (Local efficiency and Global efficiency), assortativity coefficient (α) and hierarchy coefficient (β) in functional brain networks at two different frequency bands (slow-5: 0.01–0.027 Hz, slow-4: 0.027–0.073 Hz) in case of WOGR-PAR as a function of sparsity thresholds. Local and global efficiency of random and regular networks with the same number of nodes and edges as the real networks were shown in gray lines in the network efficiency plots. (TIF) [file pone.0032766.s004.tif]

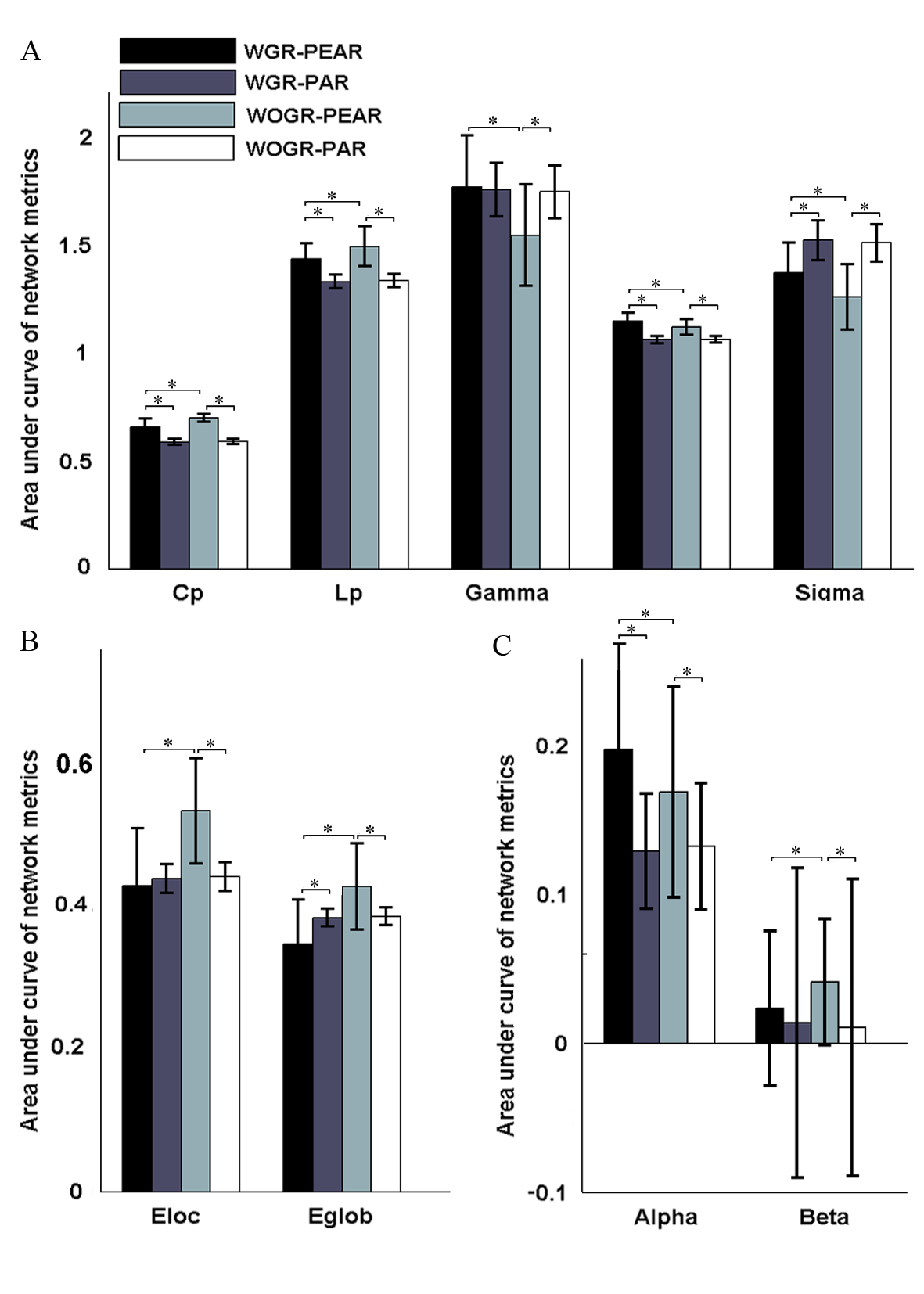

Supplement: Figure S5 — Correlation metrics and global signal dependent differences in global network properties in weighted networks. Bars show the differences in the areas under curves (AUC) of (A) small-world parameters (Cp, Lp, γ, λ and σ), (B) network efficiency (Local efficiency and Global efficiency) and (C) assortativity coefficient (α) and hierarchy coefficient (β). Error bars correspond to standard deviation of the mean across participants. The asterisk indicates p<0.05. (TIF) [file pone.0032766.s005.tif]

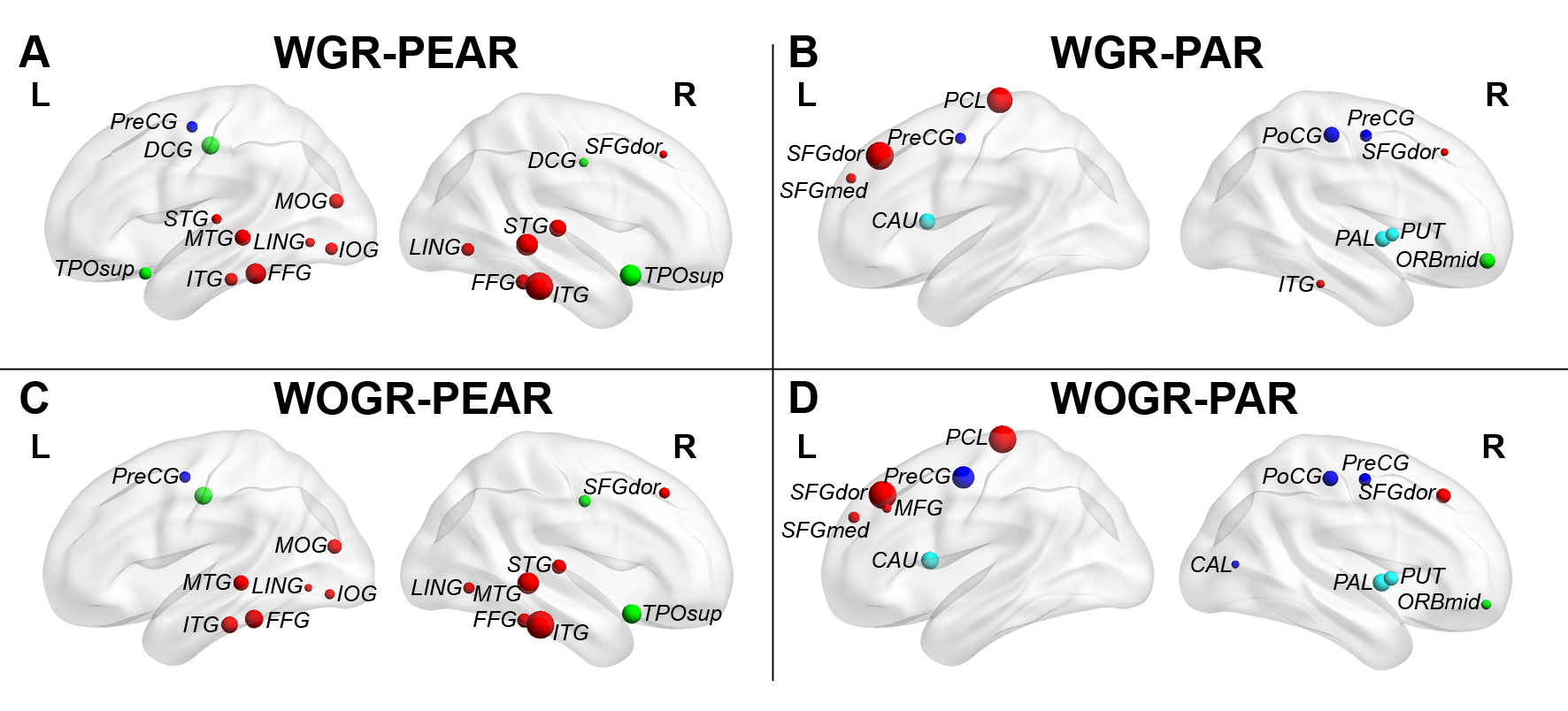

Supplement: Figure S6 — Functional hubs derived from weighted networks using different correlation metrics and global signal strategies. (A) WGR-PEAR, (B) WOGR-PEAR, (C) WGR-PAR and (D) WOGR-PAR. Regions with degree>the mean+standard deviation were considered hubs. Node colors were coded according to their membership of classical cortex classifications: association cortex (red), limbic cortex (purple), paralimbic cortex (green), subcortical regions (light blue) and primary cortex regions (dark blue). (TIF) [file pone.0032766.s006.tif]

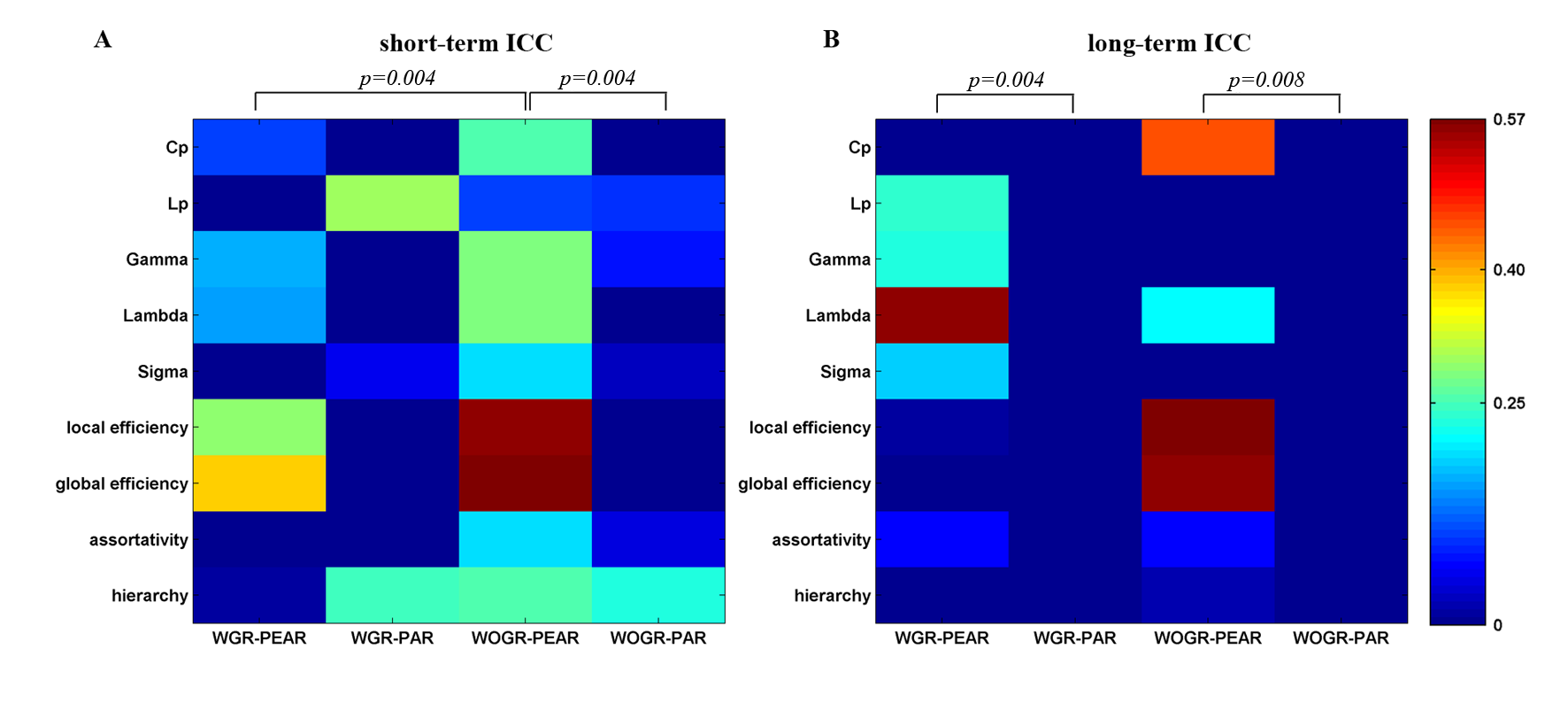

Supplement: Figure S7 — TRT reliability of global topological properties for Pearson's-correlation and partial-correlation-based weighted networks with and without global signal regression. The reliability was estimated using areas under curves (AUC) of each metric. Statistical analysis revealed significant differences in (A) short-term and/or (B) long-term TRT reliability driven by correlation metrics and/or global signal regression. (TIF) [file pone.0032766.s007.tif]

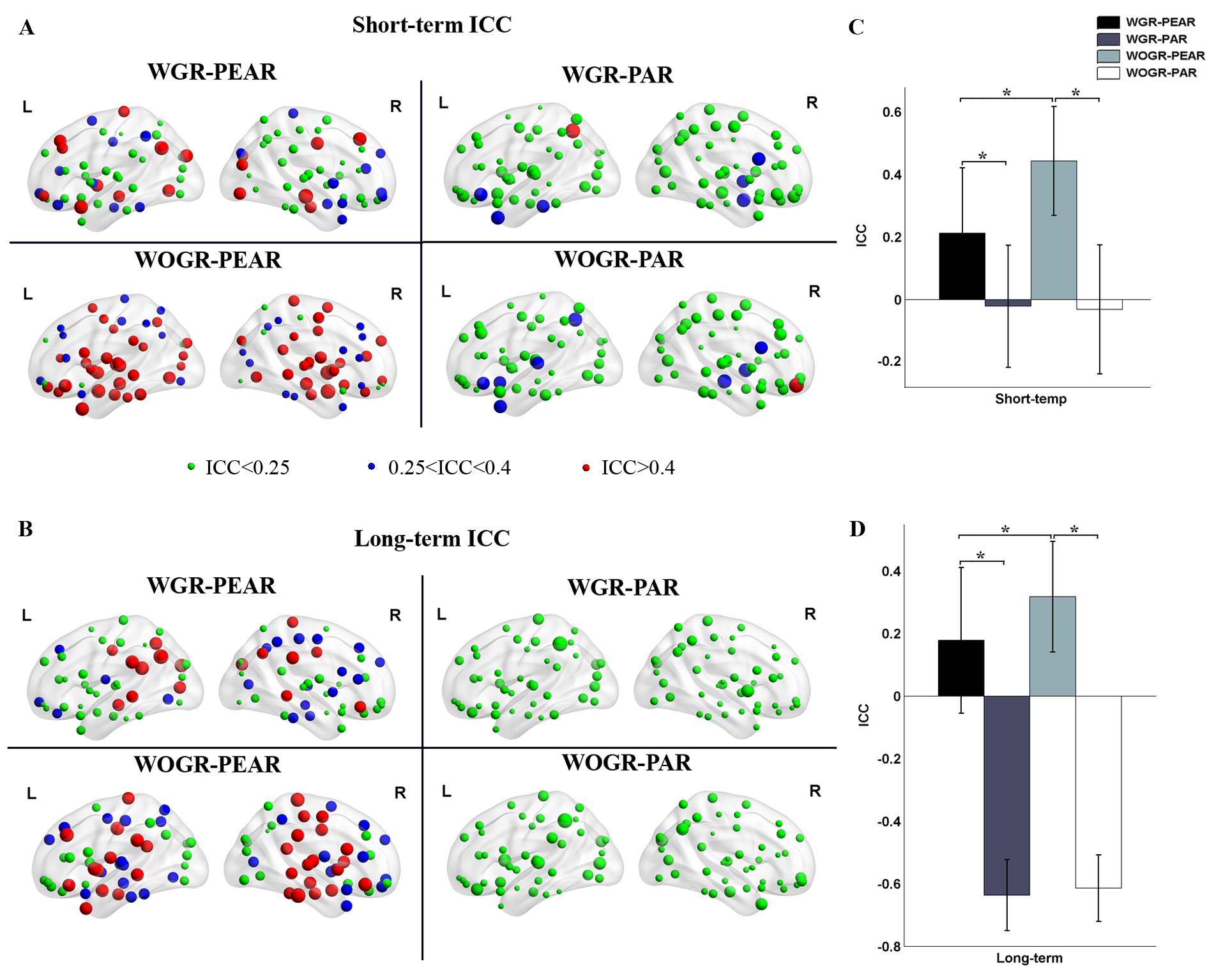

Supplement: Figure S8 — TRT reliability of nodal degree for Pearson's-correlation and partial-correlation-based weighted networks with and without global signal regression. Nodal TRT reliability values were projected onto MNI brain surface using the BrainNet viewer (http://www.nitrc.org/projects/bnv/) for (A) short-term scans and (B) long-term scans in WGR-PEAR networks, WGR-PAR networks, WOGR-PEAR networks and WOGR-PAR networks. Significant differences were found in TRT reliability of nodal degree driven by correlation metrics (Pearson's correlation/partial correlation) and global signal regression (with/without) for (C) short-term and (D) long-term scans. Nodal degree in WOGR-PEAR networks showed the highest ICC values. The asterisk indicates p<0.05. L, left hemisphere; R, right hemisphere. (TIF) [file pone.0032766.s008.tif]

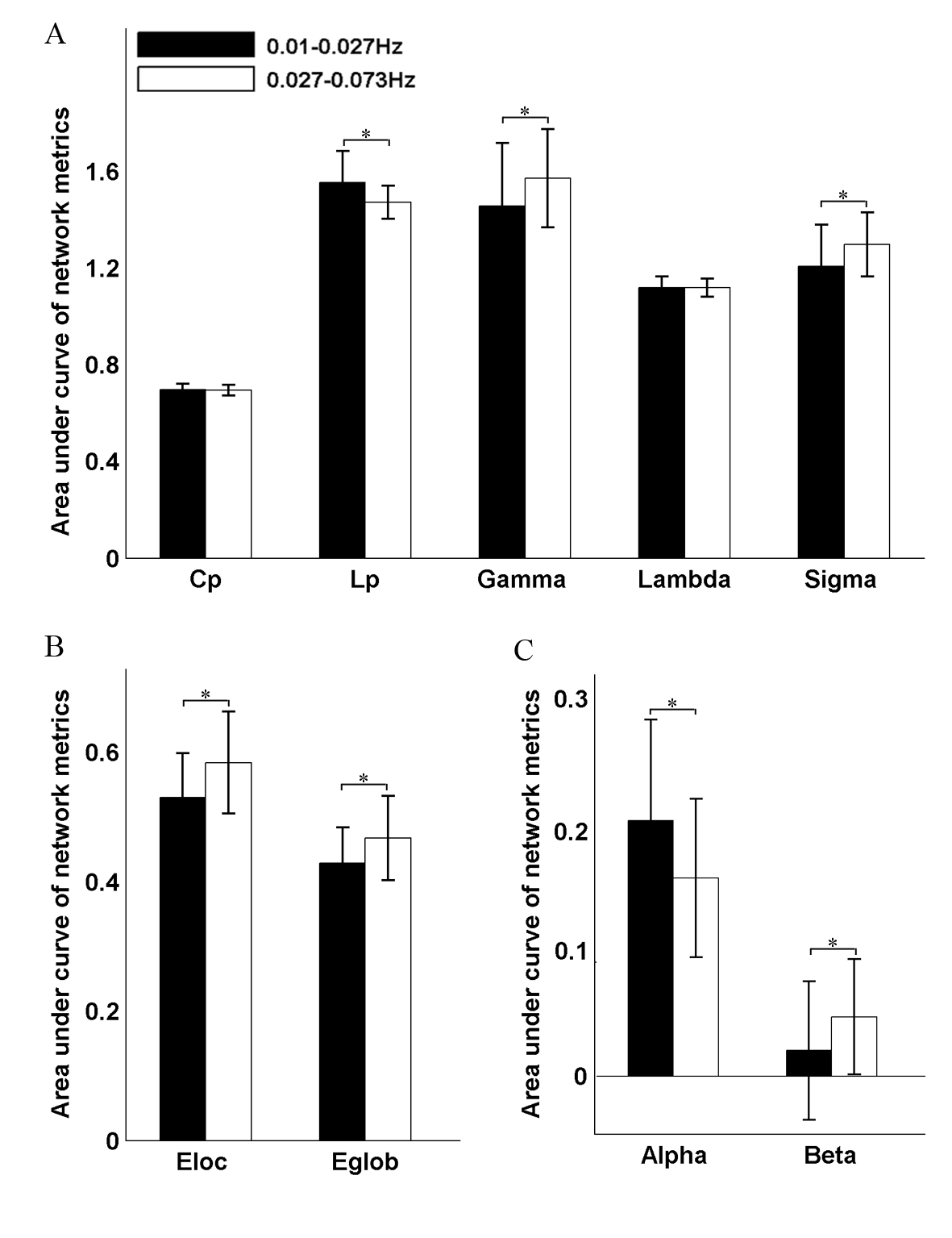

Supplement: Figure S9 — Frequency dependent differences in global network properties of weighted functional brain networks. Bars show the differences in the areas under curves (AUC) of (A) small-world parameters (Cp, Lp, γ, λ and σ), (B) network efficiency (Local efficiency and Global efficiency), (C) assortativity coefficient (α) and hierarchy coefficient (β). Error bars correspond to standard deviation of the mean across participants. The asterisk indicates p<0.05. (TIF) [file pone.0032766.s009.tif]

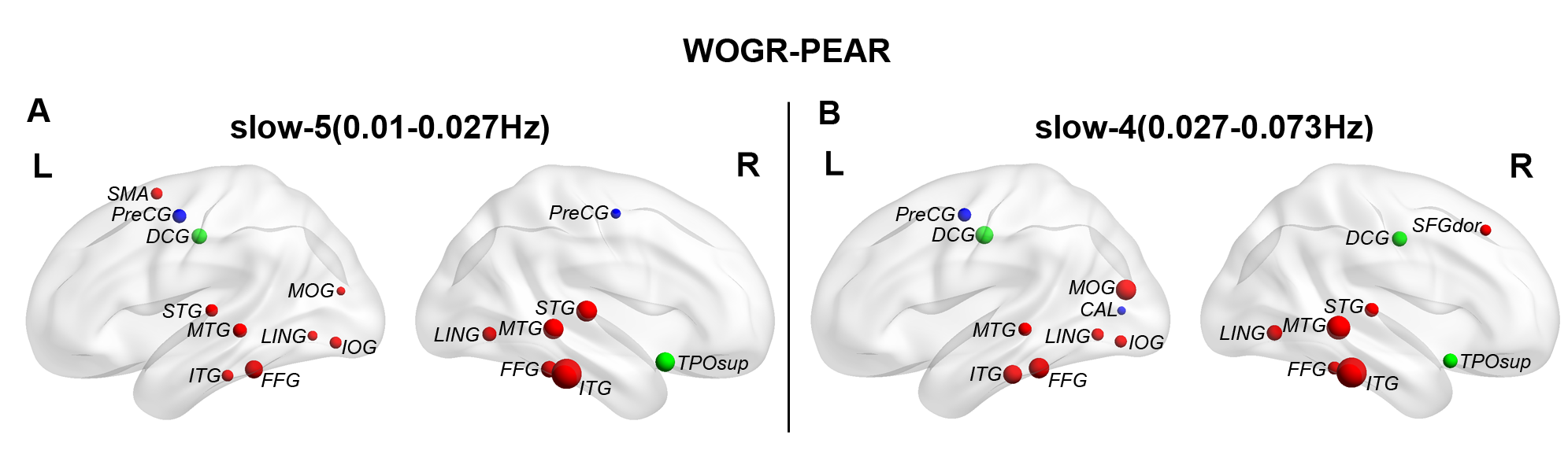

Supplement: Figure S10 — Functional hubs derived from weighted networks in different frequency bands. Regions with degree>the mean+standard deviation are considered to be hubs. Node colors were coded according to their membership of classical cortex classifications: association cortex (red), limbic cortex (purple), paralimbic cortex (green), subcortical regions (light blue) and primary cortex regions (dark blue). (A) slow-5 (0.01–0.027 Hz). (B) slow-4 (0.027–0.073 Hz). (TIF) [file pone.0032766.s010.tif]

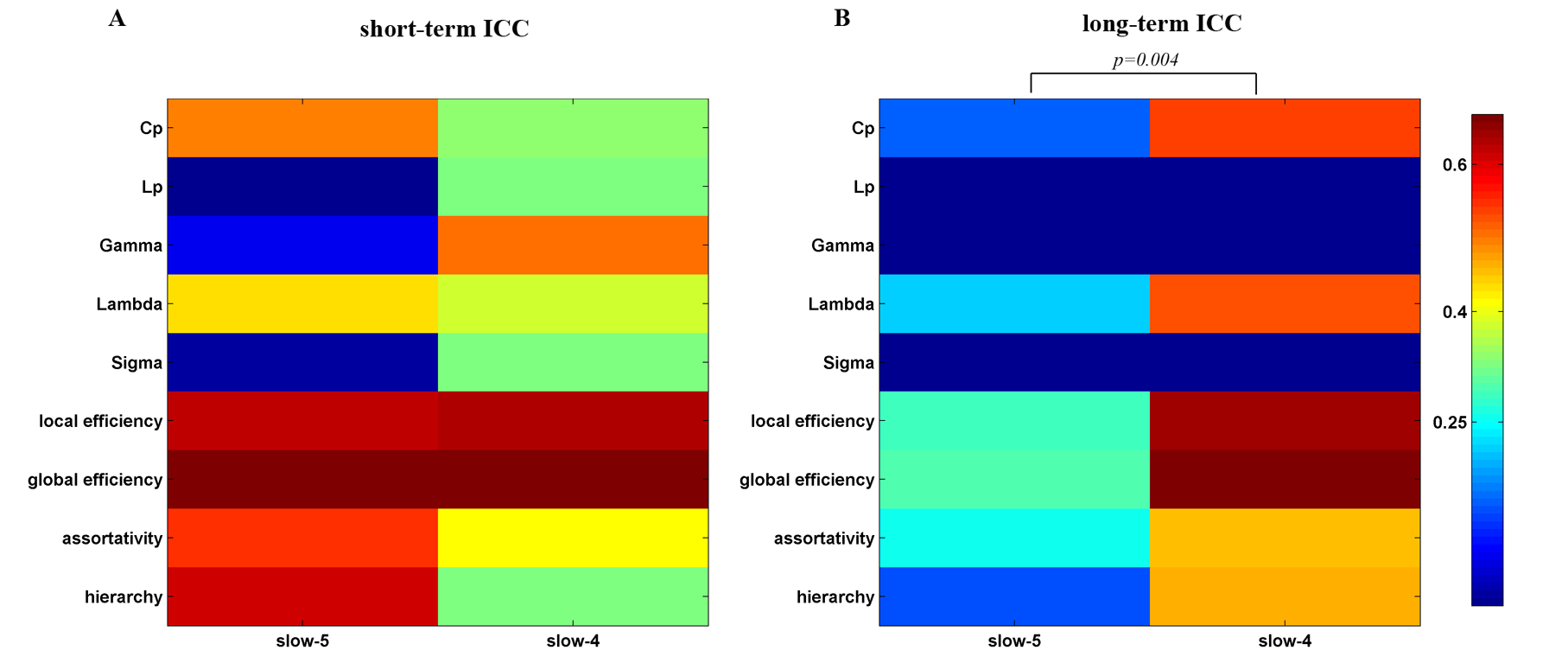

Supplement: Figure S11 — TRT reliability of global topological properties for weighted networks in different frequency bands. The reliability was estimated using areas under curves (AUC) of each metric. Statistical analysis revealed significant differences in (A) short-term and/or (B) long-term TRT reliability driven by different frequency bands. (TIF) [file pone.0032766.s011.tif]

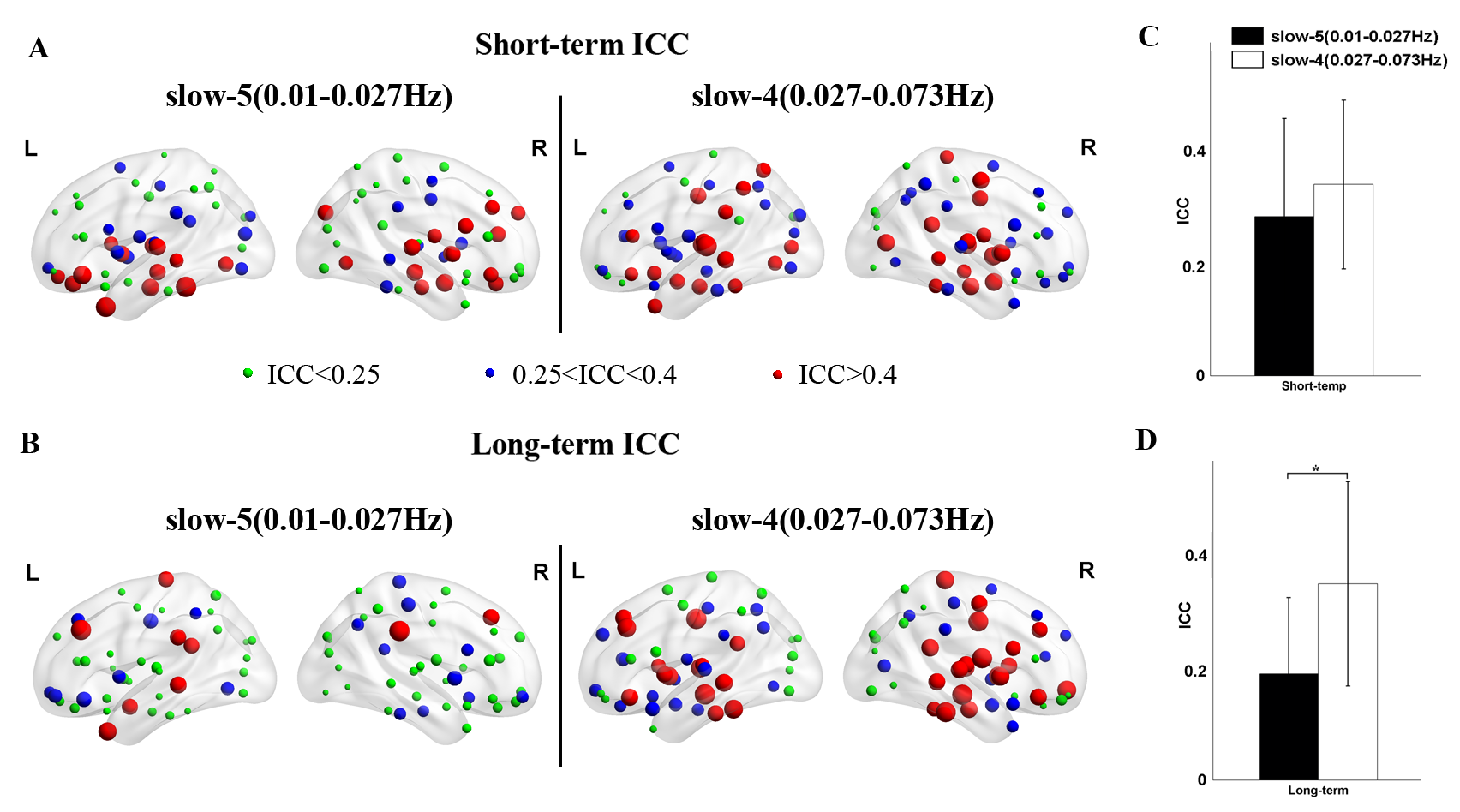

Supplement: Figure S12 — TRT reliability of nodal degree for weighted brain networks in different frequency bands. TRT reliability values of nodal degree were projected onto MNI brain surface using the BrainNet viewer (http://www.nitrc.org/projects/bnv/) for (A) short-term scans and (B) long-term scans in slow-5 and slow-4. Significant differences were found in TRT reliability of nodal degree driven by different frequency bands for (C) short-term and (D) long-term scans. Nodal degree in brain networks in slow-4 band showed higher ICC values. The asterisk indicates p<0.05. L, left hemisphere; R, right hemisphere. (TIF) [file pone.0032766.s012.tif]
