# Supplementary material for: Effects of Different Correlation Metrics and Preprocessing Factors on Small-World Brain Functional Networks: A Resting-State Functional MRI Study
Source: PLoS One. 2012 Mar 6;7(3):e32766. doi: 10.1371/journal.pone.0032766 (PMC3295769; doi:10.1371/journal.pone.0032766)
Supplement: Table S1 — Regions of interest from AAL atlas. The regions are listed in terms of a prior template of Anatomical Automatic Labeling atlas (Tzourio-Mazoyer et al., 2002). (DOC) [file pone.0032766.s013.doc]

**Supplemental Tables**

**Table S1.** Regions of interest from AAL altas.

| **Regions** | **Abbreviations** | **Regions** | **Abbreviations** |
| --- | --- | --- | --- |
| Precentral gyrus | PreCG | Lingual gyrus | LING |
| Superior frontal gyrus (dorsal) | SFGdor | Superior occipital gyrus | SOG |
| Orbitofrontal cortex (superior) | ORBsup | Middle occipital gyrus | MOG |
| Middle frontal gyrus | MFG | Inferior occipital gyrus | IOG |
| Orbitofrontal cortex (middle) | ORBmid | Fusiform gyrus | FFG |
| Inferior frontal gyrus (opercular) | IFGoperc | Postcentral gyrus | PoCG |
| Inferior frontal gyrus (triangular) | IFGtriang | Superior parietal gyrus | SPG |
| Orbitofrontal cortex (inferior) | ORBinf | Inferior parietal lobule | IPL |
| Rolandic operculum | ROL | Supramarginal gyrus | SMG |
| Supplementary motor area | SMA | Angular gyrus | ANG |
| Olfactory | OLF | Precuneus | PCUN |
| Superior frontal gyrus (medial) | SFGmed | Paracentral lobule | PCL |
| Orbitofrontal cortex (medial) | ORBmed | Caudate | CAU |
| Rectus gyrus | REC | Putamen | PUT |
| Insula | INS | Pallidum | PAL |
| Anterior cingulate gyrus | ACG | Thalamus | THA |
| Middler cingulate gyrus | MCG | Heschl gyrus | HES |
| Posterior cingulate gyrus | PCG | Superior temporal gyrus | STG |
| Hippocampus | HIP | Temporal pole (superior) | TPOsup |
| Parahippocampal gyrus | PHG | Middle temporal gyrus | MTG |
| Amygdala | AMYG | Temporal pole (middle) | TPOmid |
| Calcarine cortex | CAL | Inferior temporal gyrus | ITG |
| Cuneus CUN |  |  |  |

The regions are listed in terms of a prior template of Anatomical Automatic Labeling atlas

(Tzourio-Mazoyer et al., 2002).
