# Supplementary material for: Effects of Different Correlation Metrics and Preprocessing Factors on Small-World Brain Functional Networks: A Resting-State Functional MRI Study
Source: PLoS One. 2012 Mar 6;7(3):e32766. doi: 10.1371/journal.pone.0032766 (PMC3295769; doi:10.1371/journal.pone.0032766)
Supplement: Text S1 — (DOC) [file pone.0032766.s014.doc]

**Supplemental Materials**

**Supplemental Text**

We performed network analysis on both binary networks $A=[a_{ij}]$ and weighted networks$W=[w_{ij}]$. Binary and weighted networks were generated from correlation matrices $C=[c_{ij}]$as follows:

or

Given some network metrics are differently defined between binary and weighted networks (e.g., clustering coefficient), for these metrics, we assigned them with superscripts B or W to differentiate binary and weighted networks, respectively. Of note, for weighted network analysis, the weights were normalized by the mean weight of all non-zero elements to achieve the same level of overall connectivity strength across population and over time. Unless otherwise mentioned, all formulas are based on $G$= ($N$,$K$), a graph or network $G$ with $N$ nodes and $K$ edges.

*Small-world metrics.* Clustering coefficient ($C_{P}$) and characteristic path length are the two most important metrics for characterizing a network (Watts and Strogatz, 1998). The $C_{P}$ of a network is calculated by averaging the clustering coefficient of all nodes, where the clustering coefficient of a node $i$ ($C_{i}$) is defined as follows:

$C_{i}^{B}=\frac{2e_{i}}{k_{i}^{B}(k_{i}^{B}-1)}$ or $C_{i}^{W}=\frac{2}{k_{i}^{B}(k_{i}^{B}-1)}\sum_{j,k} (w_{ij}w_{jk}w_{ki})^{1/3}$

Where $k_{i}$ denotes the numbers of neighbors of node $i$ and $e_{i}$ denotes the actual number of edges among the neighbors of node$i$. The clustering coefficient quantifies the local interconnectivity of a graph, while the characteristic path length ($L_{P}$) characterizes the global reachablity of a graph and is defined as the average of the minimal number of edges for a binary network or the smallest sum of distances for a weighted network between all of the pairs of nodes. Here, the distance between any pair of nodes $i$ and $j$ is defined as $\frac{1}{w_{ij}}$because high correlation coefficient can be interpreted as short distance between regions.

The concept of a small-world network is based on the above two properties. Intermediate between regular (high $C_{P}$ and high $L_{P}$) and random (low $C_{P}$ and low $L_{P}$) networks, small-world networks with the organization of high $C_{P}$ and low $L_{P}$ allow themselves to be efficient on both local and global scales. Thus, by comparing the $C_{P}$ and $L_{P}$ of the real network to those in equivalent random networks (100 degree-matched random networks in our study), the real network is considered to be small-world if it fulfills the following conditions: $\gamma=C_{P}/C_{P\_rand}>>1$ and $\lambda=L_{P}/L_{P\_rand}\approx1$. A more convenient summary of small-worldness is: $\sigma=\gamma/\lambda$, which is typically more than 1 for small-world networks .

*Network efficiency.* Network efficiency, which measures the ability of information transfer of networks in terms of global efficiency and local efficiency metrics, are relative novel measurements comparing with the abovementioned conventional small-world attributes . For a graph G with N nodes, global efficiency is defined as follows:

$$E_{glob}(G)\text{=}\frac{1}{N(N\text{-}1)}\sum_{i\neq j\in G} \frac{1}{L_{i,j}}$$

Where $L_{ij}$denoting the shortest path length going from node $i$ to node$j$. And local efficiency is defined as:

$$E_{loc}(i)=\frac{1}{N_{i}(N_{i}-1)}\sum_{j\neq i\in G_{i}} \frac{1}{L_{i,j}}$$

Where $G{}_{i}$denotes the sub-graph consisting of neighbors of node$i$.

*Assortativity.* We calculated the assortativity coefficient (degree correlation) as :

$\alpha^{B}=\frac{K^{-1}\sum_{i} j_{i}k_{i}-[K^{-1}\sum_{i} \frac{1}{2}(j_{i}+k_{i})]^{2}}{K^{-1}\sum_{i} \frac{1}{2}(j_{i}^{2}+k_{i}^{2})-[K^{-1}\sum_{i} \frac{1}{2}(j_{i}+k_{i})]^{2}}$ or

$$\alpha^{W}=\frac{H^{-1}\sum_{i} \overline{w}_{i}j_{i}k_{i}-[H^{-1}\sum_{i} \frac{1}{2}\overline{w}_{i}(j_{i}+k_{i})]^{2}}{H^{-1}\sum_{i} \frac{1}{2}\overline{w}_{i}(j_{i}^{2}+k_{i}^{2})-[H^{-1}\sum_{i} \frac{1}{2}\overline{w}_{i}(j_{i}+k_{i})]^{2}}$$

Where $j_{i}$,$k_{i}$ are the degrees of the vertices at the ends of the $i$th edge, with $i=1,...,K$, and $\overline{w}_{i}$ and $H$are the weight of the $i$th edge and the total weight of all $K$edges, respectively. Nodes that have many connections (hubs) tend to be connected to other highly connected nodes in assortative networks but prefer to link with rarely connected nodes in disassortative networks.

*Hierarchy.* The hierarchical topology is a very common organization in complex networks. It is quantified by$\beta$, the exponent of the power-law relationship between clustering C and degree k of the nodes in the network :$C\sim k^{-\beta}$. If , it means that the clustering coefficient of nodes in this network decreases with the increase of their degree. This implies that the hubs prefer to link to nodes which are unlikely to be clustered with each other. Therefore, small clusters are jointed by the hubs into a single, integrated network, and the hierarchical organization is thus formed.

*Nodal degree.* The degree of node *i* is defined as:

$k_{i}^{B}=\sum_{j\in G} a_{ij}$ or $k_{i}^{W}=\sum_{j\in G} w_{ij}$

where $a_{ij}$ ($w_{ij}$) is the $(i,j)$th element in the binairzed (weighted) network of $A$($W$). Degree is a simple measurement of connectivity of a node with the rest of nodes in a network.

**References**

1. Watts DJ, Strogatz SH (1998) Collective dynamics of 'small-world' networks. Nature 393: 440-442.

2. Humphries MD, Gurney K, TJ P (2006) The brainstem reticular formation is a small world, not scale-free, network. Proc R Soc Lond B Biol Sci 273: 503--511.

3. Latora V, Marchiori M (2001) Efficient behavior of small-world networks. Phys Rev Lett 87: 198701.

4. Newman MEJ (2002) Assortative Mixing in Networks. Physical Review Letters 89: 208701.

5. Newman MEJ (2003) Mixing patterns in networks. Physical Review E 67: 026126.

6. Ravasz E, Barabási A-L (2003) Hierarchical organization in complex networks. Physical Review E 67: 026112.
